# Novel Polyelectrolytes Obtained by Direct Alkylation and Ion Replacement of a New Aromatic Polyamide Copolymer Bearing Pyridinyl Pendant Groups

**DOI:** 10.3390/polym13121993

**Published:** 2021-06-18

**Authors:** Sebastián Bonardd, Alejandro Ángel, Ángel Norambuena, Deysma Coll, Alain Tundidor-Camba, Pablo A. Ortiz

**Affiliations:** 1 Departamento de Química Orgánica, Universidad de La Laguna, Avda, Astrofísico Francisco Sánchez 3, 38206 La Laguna, Spain; s.bonardd.salvador@gmail.com; 2 Instituto de Bio-Orgánica Antonio González, Universidad de La Laguna, Avda, Astrofísico Francisco Sánchez 2, 38206 La Laguna, Spain; 3 Advanced Polymer Synthesis Laboratory (APSL), Centro de Nanotecnología Aplicada, Facultad de Ciencias, Universidad Mayor, Santiago 8580745, Chile; alejandro.angel@mayor.cl (A.Á.); angel.norambuena@mayor.cl (Á.N.); 4 Instituto de Investigaciones y Control del Ejército de Chile (IDIC), Santiago 8370899, Chile; 5 Advanced Polymer Synthesis Laboratory (APSL), Núcleo de Química y Bioquímica, Facultad de Estudios Interdisciplinarios, Universidad Mayor, Santiago 8580745, Chile; deysma.coll@umayor.cl; 6 Research Laboratory for Organic Polymers (RLOP), Department of Organic Chemistry, Pontificia Universidad Católica de Chile, Santiago 7820244, Chile; atundido@uc.cl

**Keywords:** polyamide, polyelectrolyte, pyridinyl groups, quaternisation

## Abstract

The following work shows, for the first time, the synthesis and characterization of a new family of polyelectrolytes, along with their preliminary assessments in terms of desalin water treatment. These materials fall into the category of aromatic co-polyamides, which are obtained by the direct condensation of monomers 4,4′-oxydianiline (ODA), isophthaloyl chloride, and 3,5-diamino-*N*-(pyridin-4-ylmethyl)benzamide (PyMDA). Thereby, the charged nature exhibited by these materials was achieved through the quaternization of PyMDA moieties using linear iodoalkanes of different lengths (C_n_I with *n* = 1, 2, 4, and 6). After completing the quaternization process, polyelectrolytes were subjected to a one-step anion substitution process, where iodide counterions were replaced by bis(trifluoromethane)sulfonamide entities. For all the obtained materials, solubility tests were carried out, showing that those alkylated with methyl and ethyl chains exhibit high solubility in rutinary aprotic polar solvents, while those containing *n*-butyl and *n*-hexyl units resulted in the formation of insoluble gels. Due to the above, the latest were discarded from this study early on. The structural characterization of the initial neutral co-polyamide was carried out by means of infrared spectroscopy (FT-IR), nuclear magnetic resonance (^1^H, ^13^C-NMR), and size-exclusion chromatography (SEC), while the structure of methylated and ethylated polyelectrolytes was successfully confirmed through FT-IR, ^1^H, ^13^C, and ^19^F-NMR. Additionally, the thermal behavior of these materials was analyzed in terms of thermogravimetric analysis (TGA) and differential scanning calorimetry (DSC), showing thermal degradation temperatures above 300 °C and glass transition temperatures (Tg) above 200 °C, resulting in polymers with outstanding thermal properties for water treatment applications. On the other hand, through the solvent-casting method, both neutral and charged polymers were found to be easily prepared into films, exhibiting a remarkably flexibility. The mechanical properties of the films were analyzed using the traction test, from which tensile strength values ranging between 83.5 and 87.9 Mpa, along with Young’s modulus values between 2.4 and 2.5 Gpa were obtained. Moreover, through contact angle measurements and absorption analysis by immersion, polyelectrolytes showed important changes in terms of affinity against polar and polar substances (water, *n*-heptane, and benzene), exhibiting a higher rejection regarding the neutral polymer. Finally, as a preliminary test against the seepage of saline waters, thin polymer films (from 11.4 to 17.1 µm) were deposited on top of commercial filter discs and tested as filters of saline solutions ([NaCl] = 1000 and 2000 ppm). These tests revealed a decrease of the salt concentration in the obtained filtrates, with retention values ranging between 6.2 and 20.3%, depending on the concentration of the former solution and the polymer used.

## 1. Introduction

In recent years, the rapid growth of the population and its constant demand for various resources such as energy, water, air, and food has generated a strong global concern [1,2,3,4]. This has led to the search for new methods and materials that allow obtaining these resources in more efficient and environmentally friendly ways. Among the most demanded resources, water is one of the most important, due to the important role it plays in various production processes and above all because of its importance in domestic use, being an indispensable resource for life [5,6,7].

Among the many ways to obtain suitable water for industrial and domestic use, membrane filtration processes are one of the most used due to their simplicity, smaller size, and low economic and energy costs [8]. One of the critical aspects of the filtration processes is the nature of the membrane employed, since it depends on the type of particle to be separated [9]. Regardless of the type of filtration (micro, ultra, nano, or reverse osmosis), the membrane clogging process, known as fouling, is one of the biggest problems to be solved. Regardless of the type of fouling, the clogging of the membrane reduces the production and quality of water, at the same time that production costs are increased due to the increase in the energy necessary to generate the flow of water through it [10]. Due to this, many research groups focus their works on searching for new materials that allow obtaining membranes that prevent fouling, improving the efficiency and quality of the water produced.

Within the many commercial membranes that exist today, thin-film membranes used in reverse osmosis processes are one of the most efficient due to their ability to retain particles down to one nm, making this type of membrane highly employed in desalination and water purification processes [11,12]. The above-mentioned materials are made up of two layers. The first one is a support layer generally made of polyethersulfone [13,14], while the second corresponds to a thin film or layer of cross-linked aromatic polyamide, which is usually obtained from trimesoyl chloride and *m*-phenilendiamine [15,16]. The latter is the active layer, which is responsible for separating the smaller particles from water molecules.

Within the many advantages of using aromatic polyamides in these processes, are their outstanding mechanical, thermal, and chemical resistance [17] which, together with their chemical and inner structure, allows a high flow of water through it, at the same time as it avoids the passage of the high content of salts that the feed waters present [18]. Although this type of polymers has been shown to be efficient for this kind of application, they are not exempt from problems such as fouling [19] and membrane degradation. Regarding this last issue, it has been demonstrated that polyamides suffer from oxidation phenomena after prolonged exposure to chlorides [20], which are always present at high content in waters. On the other hand, the lack of homogeneity in terms of surface/inner morphology and roughness during membrane preparation must be controlled since both factors are considered crucial in the filtration process [21,22]. Thereby, the obtainment of new aromatic polyamides capable of resisting diverse types of fouling and environments, while maintaining a high-flow water and high salt reject is of great interest. However, the main problem of aromatic polyamides is their low solubility and high Tg, which is usually ascribed to their high aromatic content and the presence of strong interactions taking place in their inner structure, such as hydrogen bonds and *π–π* stacking. It is well known that low solubilities, along with high Tg values, affect a material’s processability directly [23]. To solve this, a great number of research works have demonstrated that the inclusion of bulky groups to the polymer backbone of aromatic polyamides allows increasing the free volume inside the material by reducing the packing degree between chains, which facilitates the penetration of solvent molecules inside the material, and therefore, its solubilization [24,25]. Similarly, regarding the high Tg of these materials, it has been demonstrated that the presence of oxyether bonds in the main chain increases its flexibility, lowering the Tg value. Thereby, by manipulating the chemical structure of this type of polymers, a noticeable improvement in their processability can be achieved [26,27,28].

On the other hand, polyelectrolytes have recently acquired great interests. The attractive properties such as ionic conductivity, chemical stability, low toxicity, wide electrochemical window, and high mechanical stability that these materials present and the large number of possible applications such as desalination, membrane fouling control, pervaporation, lithium battery, fuel cells, and organic solvent nanofiltration [29,30] make them a focus of study to solve the problems that today afflict society. Among the polyelectrolytes, those that present high mechanical properties are of special interest, since they allow the transfer of the properties of an electrolyte to materials capable of forming fibers, wires, sheets, and membranes, among others, which makes polyelectrolytes versatile and multifunctional materials [31]. Among the most promising, those obtained by the alkylation of imidazole and pyridine groups attract attention, since many of them have managed to transfer the properties of ionic liquids to these polyelectrolytes, generating the family of so-called poly(ionic liquids), which correspond to liquid PEs to temperatures below 100 °C [32,33], but one of the main difficulties of these materials is their synthesis. Although there are several ways to obtain PE, these can be grouped into three methods: synthesis from ionic monomers (organic salts or ionic liquids), formation during polymerization (ionenes), or by the chemical modification of uncharged polymers (protonation–deprotonation, quaternization, or the addition of organic salts or ionic liquids). All of them present various difficulties such as obtaining ionic monomers of high purity that allow the generation of high molecular weight polymers, as in the formation during polymerization, while the chemical modification of uncharged polymers does not always allow the total conversion of all reactive groups or centers [32,34,35].

Taking into account the aforementioned, the following work aims to show the synthesis by chemical modification and characterization of new soluble and processable PEs that can be used in the manufacture of membranes applicable in processes of purification or obtaining drinking water. These PEs were manufactured from a new co-polyamide that presents: pendant pyridinyl groups for easy alkylation, oxyether groups along the chain that favor the solubility of the polymer before and after alkylation, a thermal stability of over 400 °C, and good mechanical resistance similar or superior to various commercial polymers. The work evaluates how the thermal, mechanical, and hydrophilic properties of the material are affected after alkylation, all of which are important properties for its applicability. In addition, a preliminary test is carried out to know the permeability to the passage of water and if they are able to avoid the passage of salts.

## 2. Materials and Experimental Methodology

### 2.1. Materials

*N*,*N*-dimethylacetamide anhydrous 99.8% (DMAc Anh), *N*,*N*-dimethylacetamide 99.5% (DMAc), *N*,*N*-dimethylformamide 99.5% (DMF), dimethylsulfoxide 99.0% (DMSO), dimethylsulfoxide-*d*_6_ 99.8% (DMSO-*d*_6_), *N*-methyl-2-pyrrolidinone 99.5% (NMP), 3,5-dinitrobenzoyl chloride 98.0%, 4,4′-oxydianiline 98.0% (ODA), Pd/C (10% *w*/*w*), pyridin-4-ylmethanoamine 98.0%, isophthaloyl chloride 98.0%, methyl iodide 98.0%, ethyl iodide 99%, buthyl iodide 99.0%, and hexyl iodide 99% were obtained from Sigma-Aldrich. Ethanol absolute 99.5%, *n*-hexane 99.5%, diethyl ether 99.5%, tetrahydrofuran 99.5% (THF), lithium bis(trifluoromethanesulfonyl)imide 98.0% (LiTFSI), and hydrazine (80% solution in water) were obtained from Merck. All solvents were purified by distillation under reduced pressure, except for DMAc Anh and DMSO-*d*_6_, which were used directly, as were the liquid reagents. The chlorides used were recrystallized twice from *n*-hexane, and ODA was sublimated at 300 °C under vacuum (4 × 10^−4^ mbar).

### 2.2. Experimental Part

#### 2.2.1. Characterization Techniques

FT-IR-ATR (ZnSe) were recorded on a Spectrum Two (Perkin Elmer) spectrophotometer over the range of 4000–600 cm^−1^. ^1^H, ^13^C, ^19^F, and dept 135° NMR spectra were carried out on a 400 Hz instrument (Bruker Avance III HD-400) using DMSO-*d_6_* as solvent and TMS as internal standard. Viscosimetric measurements were made in a Desreux–Bischof-type dilution viscosimeter at 30 °C (c = 0.5 g dL^−1^). The thermal behavior of polymers was studied in terms of thermogravimetric (TGA) and differential scanning calorimetry analysis (DSC), using a TGA 4000 and DSC 4000 from Perkin-Elmer, respectively. Both instruments were previously calibrated by protocols established by Perkin-Elmer. TGA thermograms were recorded between 25 and 800 °C at 10 °C/min, while DSC analysis was performed following five steps: (i) from 25 to 200 °C with a heating rate of 20 °C/min; (ii) 5 min at 200 °C; (iii) from 200 to 0 °C at a cooling rate of 20 °C/min; (iv) 5 min at 0 °C; and (v) from 0 to 200 °C at a heating rate of 10 °C/min. Glass transition temperatures (Tg) were calculated from the inflection point of the thermogram obtained from the last step. Both TGA and DSC measurements were performed under N_2_ flow (20 mL/min). The molecular weight of the polymer was determined by size-exclusion chromatography (SEC) using a Viscotek VE 1122 solvent delivery system with a VE 7510 GPC degasser and Viscotek VE 3508 differential refractive index (R.I.) detector coupled to three Viscotek LT4000L mixed columns. *N*,*N*-dimethylformamide was used as eluent (0.5 mL/min flow rate), and polystyrene standards were used for conventional calibration. Contact angle measurements were carried out using a Dataphysics OCA 20 instrument equipped with a conventional goniometer and high-performance video camera. The acquisition of the angle values was carried out using the SCA20 software. Tensile test of samples was performed under a uniaxial tension on a Shimadzu EZ-LX universal testing machine with a 1000 N load cell at a crosshead speed of 1 mm min^−1^. Films were cut in rectangular strips (10 cm × 2.5 cm) with thickness between 13 and 42 µm. The thickness of prepared samples was measured with a Fischer micrometer model DUALSCOPE MPOR, which has a measurement range of 0 to 2000 µm with a precision of 0.25 µm. A GBC atomic absorption spectrophotometer, model SavanTAA, was employed to measure NaCl concentrations.

#### 2.2.2. Synthesis of 3,5-diamino-*N*-(pyridin-4-ylmethyl)benzamide (PyMDA)

**Scheme 1:**
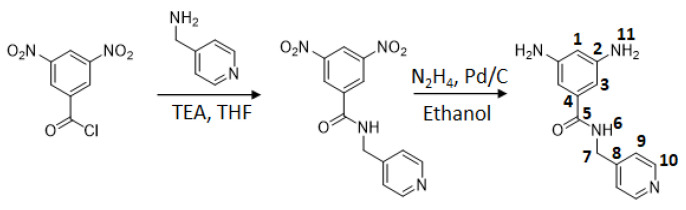
Synthesis of PyMDA diamine.

PyMDA monomer was obtained by following a two-step protocol previously reported [36]. In a first step, the direct reaction between 3,5-dinitrobenzoyl chloride and pyridin-4-ylmethanoamine takes place, allowing the obtainment of 3,5-dinitro-*N*-(pyridin-4-ylmethyl)benzamide. Then, this intermediate was exposed to a reducing environment provided by the use of Pd/C and hydrazine, allowing the obtainment of the diamine PyMDA, which was purified by sublimation at 220 °C and under vacuum a 4 × 10^−4^ mbar for later use. The PyMDA synthesis is represented in Scheme 1, and the synthetic equipment setup is shown in the Supporting Information Image S1.

PyMDA spectroscopic data. Yield: 80%. Mp: 167–169 °C. IR (KBr, cm^−1^): 3412, 3339, 3201 (N-H); 3078, 3029 (C-H, arom.); 2988, 2966, 2918 (C-H, aliph); 1632 (C=O); 1592, 1535 (C=C); 709 (mono-subst). ^1^H NMR (DMSO-*d_6_*, δ, ppm): 8.67 (t, *J* = 5.7 Hz, 1H, **6**); 8.49 (d, *J* = 4.8 Hz, 2H, **10**); 7.26 (d, *J* = 4.8 Hz, 2H, **9**); 6.30 (s, 2H, **3**); 5.99 (s, 1H, **1**); 4.89 (s, 4H, **11**); 4.41 (d, *J* = 5.9 Hz, 2H, **7**). ^13^C NMR (DMSO-*d_6_*, δ, ppm): 168.2 (**5**); 149.5 (**10**); 149.2 (**2**); 149.1 (**8**); 136.0 (**4**); 122.1 (**9**); 102.3 (**1**); 102.1 (**3**); 41.6 (**7**).

#### 2.2.3. Polymer and Polyelectrolytes Preparation

**Scheme 2:**
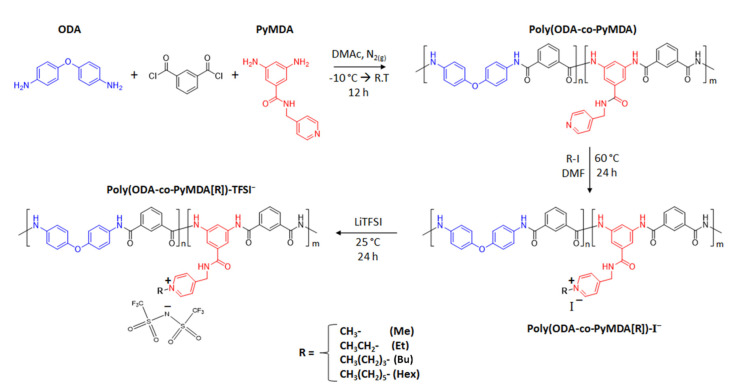
Synthesis route for obtaining poly(ODA-co-PyMDA) and its polyelectrolytes.

The complete synthetic route employed for the preparation of the polyamide labeled as poly(ODA-co-PyMDA), along with its polyelectrolytes is shown in Scheme 2. In the following sections, each reaction step is briefly described.

Synthesis of Poly(ODA-co-PyMDA)

**Scheme 3:**
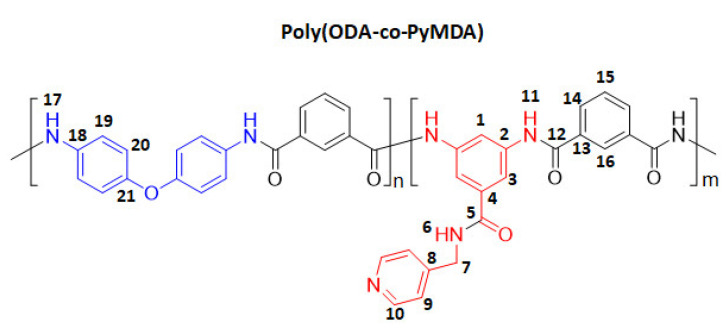
Chemical structure of poly(ODA-co-PyMDA).

In a 50 mL three-neck round-bottom flask, equipped with a mechanical stirrer and under constant N_2_ flow, 10 mmol of a 1:1 mixture of diamines ODA (1.0012 g, 5 mmol) and PyMDA (1.2115 g, 5 mmol) were completely solubilized in 10 mL of DMAc Anh. Then, 1.000 g of LiCl (5% *w*/*v*) and 5.0 mL of DMAc Anh were added to the previous dissolution under stirring until complete solubilization of the salt. The above mixture was cooled down to −10 °C using an acetone-ice bath, which was followed by adding 10 mmol of isophthaloyl chloride (2.0302 g) solubilized in 5.0 mL of DMAc Anh. The reaction was allowed to reach room temperature and kept under constant stirring and N_2_ atmosphere during 12 h. After completed the reaction time, the reaction mixture was poured into 600 mL of a 10% *w*/*v* NaHCO_3_ aqueous dissolution, and the obtained precipitated was stirred for 2 h. The polymer was filtered, washed with abundant distilled water, and purified by consecutive solubilization–precipitation cycles, using DMF and ethanol as solvent and precipitant, respectively. Finally, the polymer was cleaned in a *Soxhlet* apparatus using acetone and dried in a vacuum oven at 150 °C until constant weight. The poly(ODA-co-PyMDA) structure is represented in Scheme 3, and the synthetic equipment setup is shown in the Supporting Information Image S2A.

**Poly (ODA-co-PyMDA) spectroscopic data.** Conversion: 76%. FT-IR-ATR (ZnSe, cm^−1^): 3296 (N-H); 3064 (C-H arom.); 1656 (C=O); 1600, 1494 (C=C); 1534 (C=N); 1233, 1213 (C-N); 876, 683 (1,3-disubst); 828 (1,4-disubst); 769, 723 (mono-subst.). ^1^H NMR (DMSO-*d_6_*, δ, ppm): 10.3 (s, 2H, **11**); 10.1 (s, 2H, **17**); 8.67 (s, 1H, **6**); 8.52 (s, 1H, **1**); 8.03 (s, 2H, **3**); 7.34 (d, *J* = 4.6 Hz, 2H, **9**); 8.60 (t, *J* = 15.7 Hz, 2H, **16**); 8.51 (s, 2H, **10**); 8.18 (dt, *J* = 7.3/15.3 Hz, 4H, **14**); 7.80 (d, *J* = 8.9 Hz, 4H, **19**); 7.68 (p, *J* = 7.6 Hz, 2H, **15**); 7.06 (d, *J* = 8.8 Hz, 4H, **20**); 4.55 (d, 3.8, 2H, **7**). ^13^C NMR (DMSO-*d_6_*, δ, ppm): 166.3 (**5**); 164.7/164.4 (**12**); 152.8 (**21**); 148.9 (**10**); 147.8 (**8**); 138.8 (**2**); 135.1 (**4**); 135.0/134.9/134.7/134.7 (**13**); 134.1/134.0 (**18**); 129.9/129.8/129.7/129.6 (**16**); 127.9/127.8/127.7 (**15**); 126.4/126.3/126.2 (**14**); 121.9 (**19**); 121.7 (**9**); 118.0 (**20**); 115.8 (**1**); 115.3 (**3**), 41.6 (**7**).

Preparation of Polyelectrolytes by Quaternization of Poly(ODA-co-PyMDA) and Its Subsequent Counterion Exchange

**Scheme 4:**
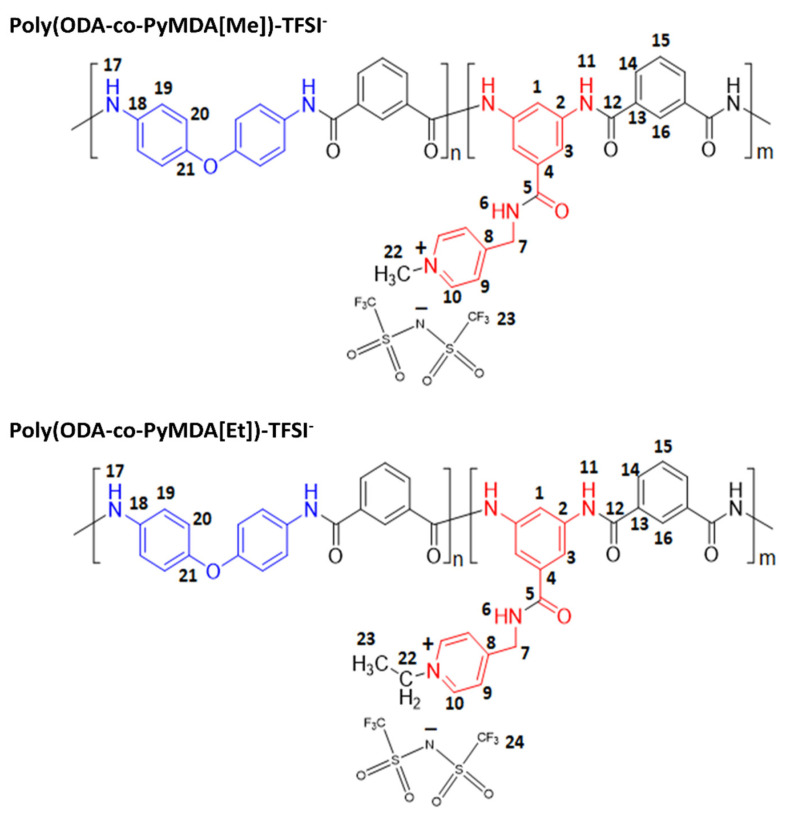
Chemical structures of polyelectrolytes derived from poly(ODA-co-PyMDA).

To a 50 mL round-bottom flask, equipped with a magnetic stirrer and condenser, 2.000 g (10% *w*/*v*) of poly(ODA-co-PyMDA) and 20 mL of DMF were mixed until achieving the complete dissolution of the polymer. Then, 1.0 mL (5% *v*/*v*) of the corresponding iodoalkane was added, allowing the reaction to proceed at 60 °C for 24 h. After completing the reaction time, the mixture was cooled down to room temperature, and 1.000 g (5% *w*/*v*) of LiTFSI was added. The above solution was kept under constant stirring for 12 h, allowing the anion-exchange process, before finally being poured into 400 mL of distilled water. The precipitated was filtered, washed with abundant distilled water, and dried at 150 °C overnight. Finally, the obtained polymer was cleaned by *Soxhlet* extraction using acetone and dried again until constant weight. It is important to mention that during the alkylation process, samples poly(ODA-co-PyMDA[Bu])-I and poly(ODA-co-PyMDA[Hex])-I resulted in the irreversible formation of insoluble gels. The results of the infrared spectroscopy analysis are shown in the Schemes S1 and S2 of the Supporting Information Due to the above, these samples were discarded since they were not able to be processed in terms of the aim of the present work. Scheme 4 exposes the chemical structures of polyelectrolytes poly(ODA-co-PyMDA[Me])-TFSI^−^ and poly(ODA-co-PyMDA[Et])-TFSI^−^, and the synthetic equipment setup is shown in the Supporting Information Image S2B.

**Poly(ODA-co-PyMDA[Me])-TFSI^−^ spectroscopic data.** Yield: 89%. FT-IR-ATR (ZnSe, cm^−1^): 3231 (N-H); 3025 (C-H arom.); 1647 (C=O); 1602, 1445 (C=C); 1534 (C=N); 1496 (N-H); 1231, 1213 (C-N); 1348, 1328 (SO_2_, Asym); 1228 (CF_3_, Asym); 1987 (CF_3_, Sym); 1131 (SO_2_; Sym); 1158 (SNS); 770, 720 (mono-subst.). ^1^H NMR (DMSO-*d_6_*, δ, ppm): 10.71/10.64 (s, 3H, **11**); 10.45 (s, 3H, **17**); 9.38 (t, *J* = 5.9 Hz, 1H, **6**); 8.87 (d, *J* = 6.6 Hz, 2H, **10**); 8.56 (m, 2H, **16**); 8.56 (m, 1H, **1**); 8.18 (m, 4H, **14**); 8.18 (m, 2H, **3**); 7.99 (d, *J* = 6.6 Hz, 2H, **9**); 7.80 (d, *J* = 9.2, 6H, **19**); 7.72 (m, 2H, **15**); 7.06 (d, *J* = 7.0, 6H, **20**); 4.75 (d, *J* = 5.7 Hz, 2H, **7**); 4.30 (s, 3H, **22**). ^13^C NMR (DMSO-*d_6_*, δ, ppm): 167.2 (**5**); 165.5/165.1 (**12**); 159.2 (**8**); 153.1 (**21**); 145.3 (**10**); 139.6 (**2**); 135.3/135.2 (**4**); 135.0 (**13**); 134.7 (**18**); 130.8 (**14**); 128.9 (**15**); 127.3 (**16**); 125.4 (**9**); 122.4 (**19**); 121.2/118.0 (**28**); 118.9 (**20**); 116,1 (**1**); 115.6 (**3**); 47.5 (**22**); 42.5 (**7**). ^19^F NMR (DMSO-*d_6_*, δ, ppm): −78.68.

**Poly(ODA-co-PyMDA[Et])-TFSI^−^ spectroscopic data.** Yield: 84%. FT-IR-ATR (ZnSe, cm^−1^): 3231 (N-H); 3025 (C-H arom.); 1647 (C=O); 1602, 1445 (C=C); 1534 (C=N); 1496 (N-H); 1233, 1213 (C-N); 1346, 1325 (SO_2_, Asym); 1228 (CF_3_, Asym); 1192 (CF_3_, Sym); 1131 (SO_2_; Sym); 1154 (SNS); 770, 720 (mono-subst.). ^1^H NMR (DMSO-*d_6_*, δ, ppm): 10.71 (s, 2H, **11**); 10.45 (s, 2H, **17**); 9.38 (t, *J* = 5.3 Hz, 1H, **6**); 9.02 (d, *J* = 5.5 Hz, 2H, **10**); 8.56 (m, 2H, **16**); 8.53 (s, 1H, **1**); 8.18 (m, 4H, **14**); 8.12 (s, 2H, **3**); 8.03 (d, *J* = 5.6 Hz, 2H, **9**); 7.81 (d, *J* = 8.1, 4H, **19**); 7.73 (m, 2H, **15**); 7.06 (d, *J* = 7.6, 4H, **20**); 4.77 (s, 2H, **7**); 4.60 (q, *J* = 7.6 Hz, 2H, **22**); 1.53 (t, *J* = 6.7 Hz, 3H, **23**). ^13^C NMR (DMSO-*d_6_*, δ, ppm): 167.1 (**5**); 165.4/165.1 (**12**); 159.5 (**8**); 153.1 (**21**); 144.2 (**10**); 139.5 (**2**); 135.2 (**4**); 135.0/134.5 (**13**); 134.6 (**18**); 131.0 (**14**); 128.9 (**15**); 127.2 (**16**); 125.8 (**9**); 122.3 (**19**); 121.2/118.0 (**28**); 118.8 (**20**); 116,1 (**1**); 115.6 (**3**); 56.0 (**22**); 42.5 (**7**). ^19^F NMR (DMSO-*d_6_*, δ, ppm): −78.68.

### 2.3. Films Preparation

Films were obtained through the solvent-casting method. Briefly, 800 mg of polymer were dissolved in 20 mL of DMF (4% *w*/*v*) and filtered through a glass fiber of 3.1 μm porosity. The filtered solutions were deposited on Petri dishes (15 cm in diameter) and kept at 30 °C for 12 h. Subsequently, formed films were peeled off from dishes and placed between two stainless-steel mesh and dried at 150 °C during 24 h. The obtained films exhibited a remarkably flexibility and thickness values ranging between 13 and 42 μm.

### 2.4. Absorption Tests

Absorption tests were carried out by immersing a rectangular piece of film (1.5 cm × 2.0 cm) into 15 mL of solvent. Films were periodically extracted, quickly dried and weighted, to then re-immersed again. This cycle was repeated for each sample until obtaining a constant mass. The absorption percentage was calculated using Equation (1):

(1) $$Abs\;\%=\frac{\left(W_{t\;}-W_{i}\right)\times 100}{W_{i}}$$

where $W_{t\;}$ refers to the mass of the film submerged at time *t*, and $W_{i}$ is the initial film mass (*t* = 0).

### 2.5. Evaluation of Membrane Performance

#### 2.5.1. Membrane Preparation

On a filter, discs grade 388 of 110 mm of diameter (filter cut-off = 10–15 µm), 4 mL of polyelectrolyte solution in DMF (0.5 g mL^−1^) were deposited and subsequently heated at 30 °C for 24 h. Then, obtained discs were immersed in methanol for 2 h, dried under vacuum at 50 °C for 24 h, and finally cut into a 5 cm diameter discs.

#### 2.5.2. Water Flux and Salt Rejection

**Scheme 5:**
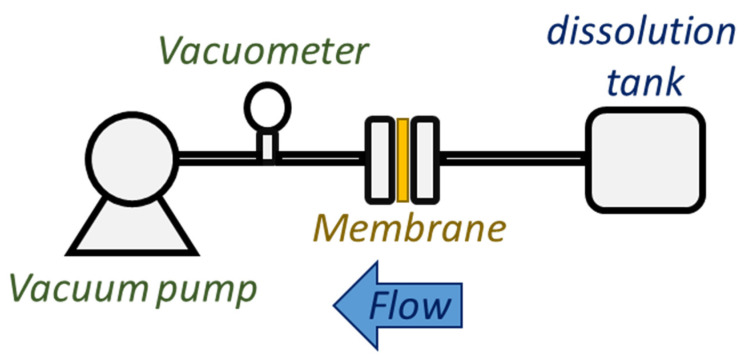
Transverse filtration system.

Tests were carried out using a transverse filtration system represented in Scheme 5. Briefly, 100 mL of NaCl solutions of two different concentrations (1000 and 2000 ppm) were passing through the above-mentioned membranes (Section 2.5.1) at 25 °C. The effective area of membranes was 9.62 cm^2^ while, depending on the case, two vacuum pressures (14 or 40 cmHg) were applied.

The flux calculations were performed using Equation (2):

(2) $$J=\frac{V}{At}$$

where *J* = water flux (L/(m^2^h)), *V* = volume (L), *A* = area (m^2^), and *t* = time (h).

On the other hand, salt rejection values (*R_(i)_*) were calculated using Equation (3):

(3) $$R_{\left(i\right)\;}=\frac{\left(C_{f}-C_{p}\right)\times 100}{C_{f}}$$

where *C_p_* (mg/L): filtering concentration, and *C_f_* (mg/L): feed concentration. Salt concentrations were determined by atomic absorption.

## 3. Result and Discussion

**Figure 1:**
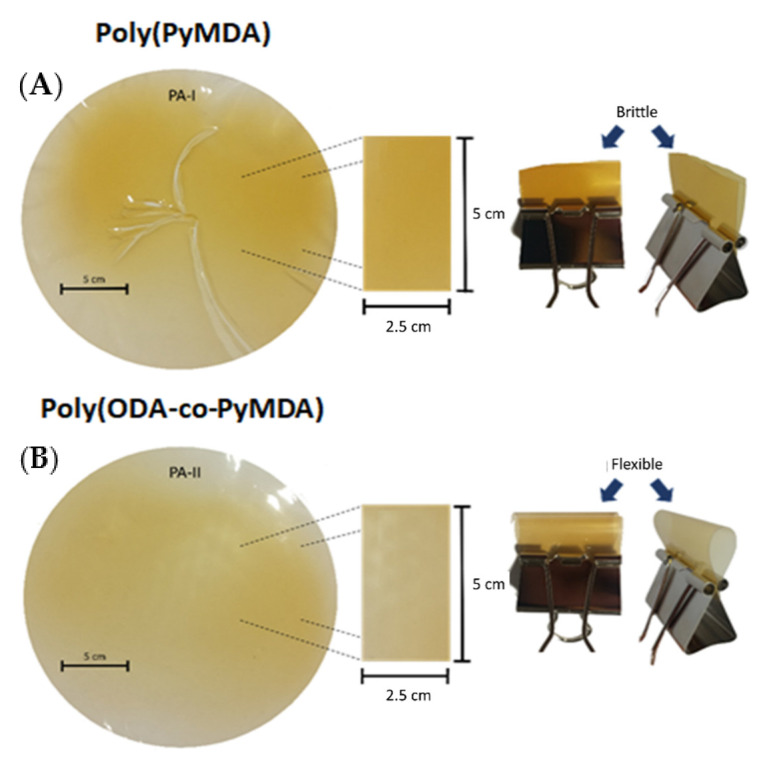
Images of films obtained by solvent-casting from poly(PyMDA) (**A**) and poly(ODA-co-PyMDA) (**B**) DMF solutions.

The present discussion will begin by presenting results regarding the synthesis and structural characterization of poly(ODA-co-PyMDA). This polyamide was successfully prepared following previously reported protocols, involving the direct coupling of homo-bifunctional aromatic monomers, in this case, diamines and diacid chlorides. Particularly, poly(ODA-co-PyMDA) could be classified as a co-polyamide, since two different diamines were used, PyMDA and ODA. The choice of both diamines was not aleatory and will be explained below. The use of PyMDA results is fundamental for the development of this work, since it would allow obtaining polyelectrolytes by the direct quaternization of its pyridinyl pendant group. On the other hand, it has been widely reported that the introduction of ODA entities helps to increase the processability of polyamides, aiming to the formation of more flexible films with improved mechanical properties. Indeed, at the very beginning of this project, the structure of the target polyamide was devised using purely PyMDA and isophthaloyl chloride. Therefore, prior to the preparation of poly(ODA-co-PyMDA), our group was focused on the synthesis of poly(PyMDA), which was successfully obtained by following the same polymerization protocol mentioned above. However, as soon as this polymer was synthesized, it not only revealed difficulties in the formation of homogeneous films, but also, the obtained films turned out to be brittle materials. The above was the main motivation to include ODA units in the structure of PyMDA-bearing polyamides, carrying out the preparation of poly(ODA-co-PyMDA) which, unlike poly(PyMDA), allowed the manufacture of more homogeneous and flexible films. The above can be verified by a simple inspection of the images shown in Figure 1.

Therefore, having argued the use of both types of diamines, the present work was based on the synthesis of poly(ODA-co-PyMDA) and its polyelectrolytes, together with the study of their properties and possible application in water treatment. On the other hand, poly(PyMDA) and poly(ODA) (also synthesized and characterized in this work, see Schemes S3 and S4 of the Support Information) were used as reference materials during the characterization of poly(ODA-co-PyMDA), and the results of our characterization (FT-IR, NMR, test solubility, inherent viscosity and TGA) can be found in the Supporting Information (Figures S1−S4 and Table S1).

Regarding the synthesis of poly(ODA-co-PyMDA), the experimental conditions employed allowed achieving a high polymerization grade, as well as a suitable degree of purity. The use of LiCl during the polymerization had been supported by several authors, explaining that the presence of this salt in the reaction media promotes the solubility of the growing polymer chains [37]. Due to the above, polymer chains remain in solution, and therefore, in contact with remnant monomers and other polymer chains, favoring the growth of chains and increasing the molecular weight of the material. In fact, our tests without LiCl showed the presence of an early precipitate during polymerizations. Another experimental parameter evaluated by us was the low reaction temperature used during the addition of isophtaloyl chloride. The above has been ascribed to the exothermic nature of the reaction, where an abrupt temperature increase could induce to the apparition of side reactions, affecting directly the polymerization process in terms of monomer conversion and purity. Once the polymerization time is completed, the reaction must be poured into an alkaline media in order to neutralize the pyridinyl moieties protonated by the HCl released during the process. Thanks to this, a more effective precipitation of the polymer was achieved. Finally, the purification method used in this work resulted in being effective, since no traces of unreacted monomers were observed in polymer samples.

**Figure 2:**
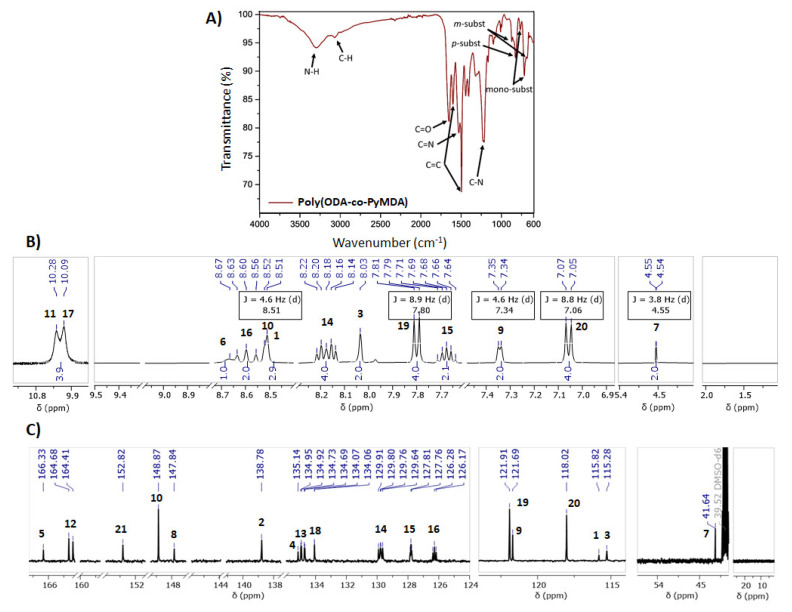
FT-IR (**A**), ^1^H (**B**), and ^13^C-NMR (**C**) spectra of poly(ODA-co-PyMDA). NMR measurements recorded in DMSO-*d*_6_.

The chemical structure of poly(ODA-co-PyMDA) was corroborated by means of FT-IR, ^1^H, and ^13^C-NMR, whose results are shown in Figure 2.

**Figure 3:**
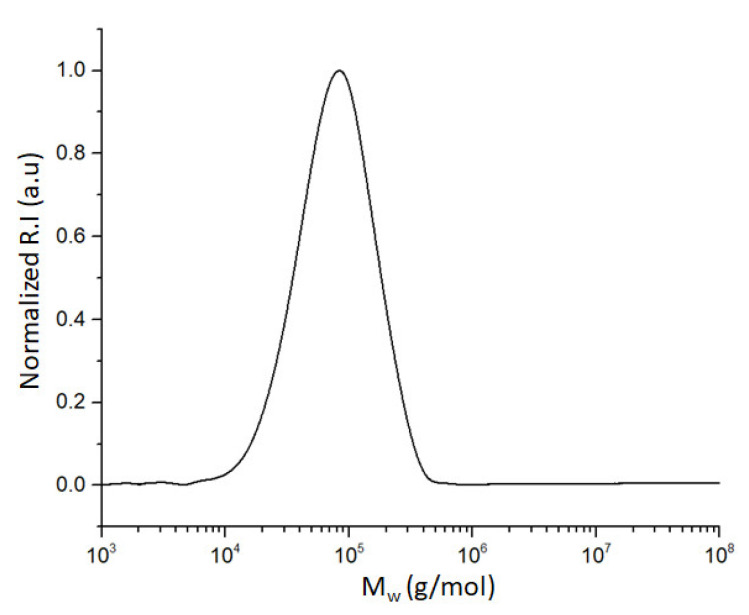
Size-exclusion chromatography (SEC) trace for poly(ODA-co-PyMDA).

From FT-IR analysis (Figure 2A), the presence of typical signals ascribed to the expected functional groups in the polymer can be identified. Within them, those corresponding to N−H and C=O structures allow the identification of amide linkages coming from the polymerization process but also from the PyMDA monomer. Both signals can be found centered at 3296 and 1656 cm^−1^, respectively. In addition, the aromatic structure of poly(ODA-co-PyMDA) can be confirmed by the presence of two vibrational bands at 1600 and 1494 cm^−1^, which has been previously assigned to C=C but also to a series of less intense signals detected in the range between 1000 and 600 cm^−1^. These last signals have been correlated to vibrational modes arising from meta and para-substituted benzene rings. Finally, signals referred to C=N and C−N bonds can be observed at 1534 and 1233 cm^−1^, respectively, where the first one would demonstrate the presence of pyridinyl pendant groups. On the other hand, Figure 2B,C exhibit a complete and successful signal assignment for the most relevant protons and carbons, corroborating the correct structure of the obtained polymer. This assignment was carried out by using as reference the NMR spectra recorded for poly(PyMDA) and poly(ODA), which are both attached in the Supporting Information File (Figures S2 and S3). Concerning the signals assignment, the ^1^H-NMR analysis allowed the identification of two signals between 11 and 10 ppm. The signal at 10.1 ppm (17) would be assigned to protons belonging to amides prepared from ODA units, while the signal at 10.3 ppm (11) should be ascribed to protons present in amide structures containing PyMDA moieties. Another important signal is the one observed at 4.5 ppm (7), representing the methylene unit present in the PyMDA structure. Lastly, high-intensity signals (doublets) labeled as 19 and 20 have been related to protons from the ODA structure. Therefore, both ODA and PyMDA structures were confirmed in the polymer backbone. Indeed, based on the integral analysis of the respective proton signals, it is shown that the ODA:PyMDA ratio present in the polymer correlates well with the initial monomer feed ratio employed during the polymerization. Based on the above, the correct chemical structure of poly(ODA-co-PyMDA) was confirmed. Finally, in order to confirm the macromolecular nature of poly(ODA-co-PyMDA), its weight-average molecular weight (M_w_), number-average molecular weight (M_n_), and dispersity (Đ) were determined by SEC analysis (Figure 3). The results are summarized in Table 1.

**Figure 4:**
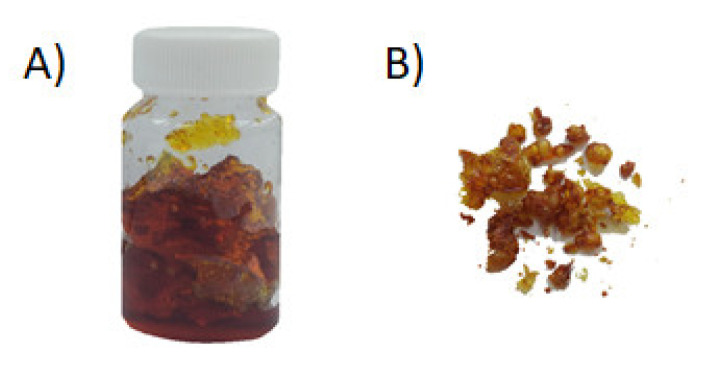
Examples of insoluble gels obtained during alkylation of poly(ODA-co-PyMDA) with butyl and hexyl iodide (**A**) and gels dried at 150 °C (**B**).

Once we completed the structural characterization of poly(ODA-co-PyMDA), the next step was to carry out the alkylation of its pyridinyl entities aiming at the preparation of polyelectrolytes (see Scheme 1). However, prior to performing the reaction over the polyamide, assays using 3,5-dinitro-*N*-(pyridin-4-ylmethyl)benzamide as a model molecule were carried out in order to determine the optimum conditions to perform the quaternization. Two types of alkylating agents (ethyl bromide and ethyl iodide) were tested, obtaining markedly better results using the iodinated reagent (date not shown). Due to the above, poly(ODA-co-PyMDA) was subsequently alkylated by using methyl iodide, ethyl iodide, butyl iodide, and hexyl iodide. It is worth noting that the alkylation of poly(ODA-co-PyMDA) with butyl and hexyl iodide gave way to the formation of insoluble gels, as is shown in Figure 4A,B, forcing them to be discarded from this work.

**Figure 5:**
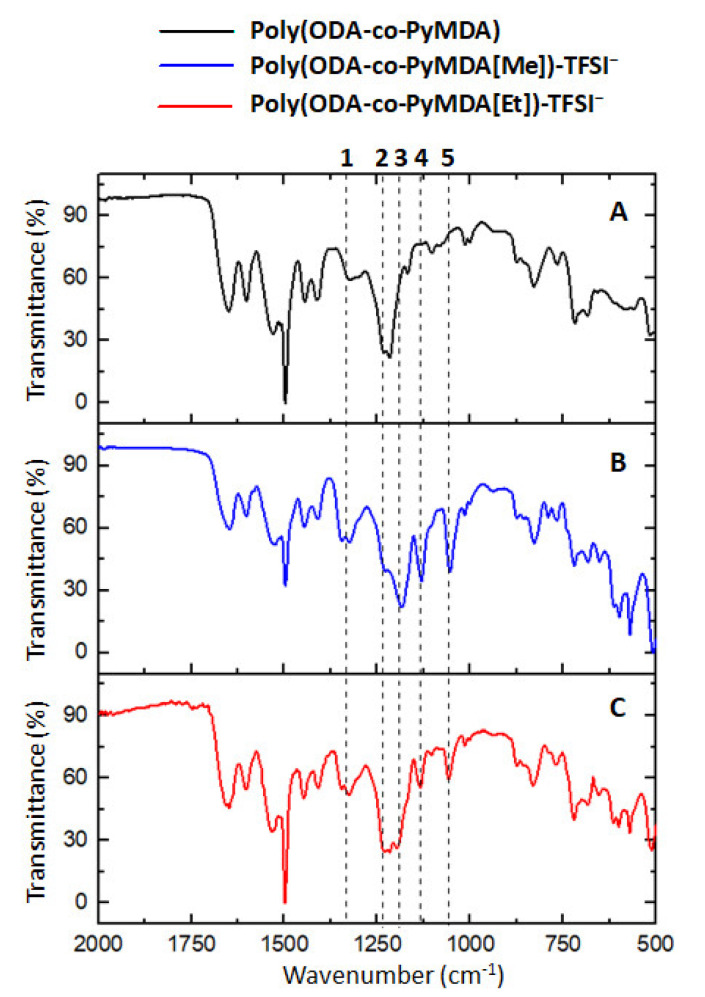
FT-IR spectra of poly(ODA-co-PyMDA) (**A**), Poly(ODA-co-PyMDA[Me])-TFSI^−^ (**B)** and poly(ODA-co-PyMDA[Et])-TFSI^−^ (**C**).

Fortunately, the polyelectrolytes obtained using methyl and ethyl iodides remained soluble, which allowed them to undergo an anion exchange process by the direct addition of LiTFSI to the reaction media. Poly(ODA-co-PyMDA[Me])-TFSI^−^ and poly(ODA-co-PyMDA[Et])-TFSI^−^ were successfully obtained as brown powders and characterized, in a first stage, by means of FT-IR, ^1^H, ^13^C, and ^19^F-NMR. Figure 5 shows a comparison between the FT-IR spectra of poly(ODA-co-PyMDA) (Figure 5A) and both polyelectrolytes (Figure 5B,C).

**Figure 6:**
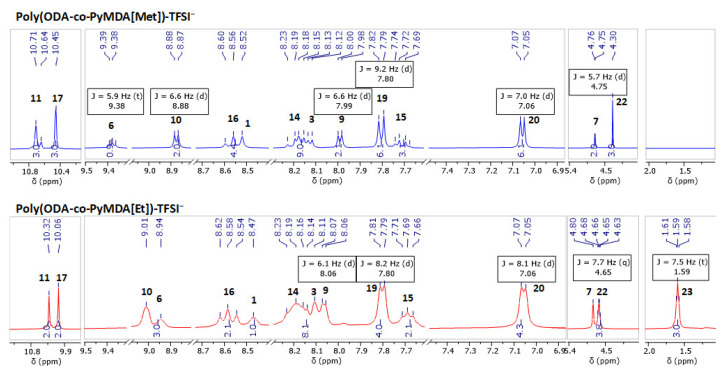
^1^H-NMR of polyelectrolytes derived from poly(ODA-co-PyMDA). NMR spectra measured in DMSO-*d_6_*.

**Figure 7:**
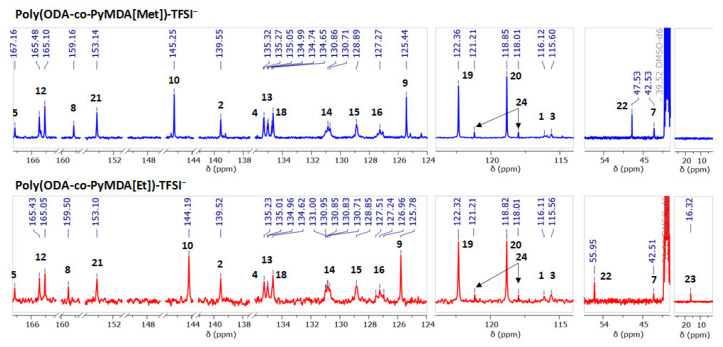
^13^C-NMR of polyelectrolytes derived from poly(ODA-co-PyMDA). NMR spectra measured in DMSO-*d_6_*.

Taking as a starting point the previously discussed FT-IR spectrum for poly(ODA-co-PyMDA), Figure 5 allows the identification of new signals appearing after the polyamide quaternization. These new signals can be ascribed to vibrational modes of bonds belonging to the TFSI^−^ counterion. In this regard, signals visualized around 1325 and 1131 cm^−1^ (labeled as 1 and 4, respectively) are related to the asymmetric and symmetric stretching vibrations of SO_2_ groups. Moreover, signals observed at 1228 cm^−1^ (2) and 1192 cm^−1^ (3) have been ascribed to the presence of CF_3_ groups vibrating asymmetrically and symmetrically, respectively. Finally, the signal centered at 1054 cm^−1^ (5) arises from the vibrational motions of S−N−S structures. Based on the above, FT-IR measurements would confirm the presence of TFSI^−^ counterions electrostatically attached to the polyamide structure. Additionally, the ^1^H and ^13^C-NMR spectra shown in Figure 6 and Figure 7, respectively, were also used as evidence of the quaternization carried out on poly(ODA-co-PyMDA).

Figure 6 reveals the successful assignment of the most relevant proton signals present in both polyelectrolytes. The assignment and analysis of both spectra were performed using as reference the spectra recorded for poly(ODA-co-PyMDA) (Figure 2B). In this sense, the apparition of new signals and the chemical shifts experienced by others confirm the correct alkylation of the polyamide. In detail, it draws attention to both polyelectrolytes, the noticeable displacement exhibited by signals 6, 9, and 10, along with the apparition of new signals labeled as 22 and 23. The displacement experienced by the first three signals mentioned above could be ascribed to changes in the electronic density of pyridinyl rings after their alkylation, which is mainly due to the inductive effect. On the other hand, the apparition of signals 22 and 23 reveals the presence of methyl and ethyl groups bonded to the quaternized nitrogen. In addition, a similar analysis was carried out to ^13^C-NMR spectra, allowing the detection and assignment of the most relevant carbon signals of the polyelectrolyte structures (Figure 7). In this regard, important displacements were observed for those carbons belonging to pyridinyl structures, along with the apparition of signals 22 and 23 related to alkylating agents. However, in addition to the above signals, two new signals pointed out by black arrows in Figure 7 and labeled as 24 appear in the spectra of both polyelectrolytes at 121.21 and 118.01 ppm. These signals have been assigned to CF_3_ structures and, therefore, caused by the presence of TFSI^−^ counterions.

**Figure 8:**
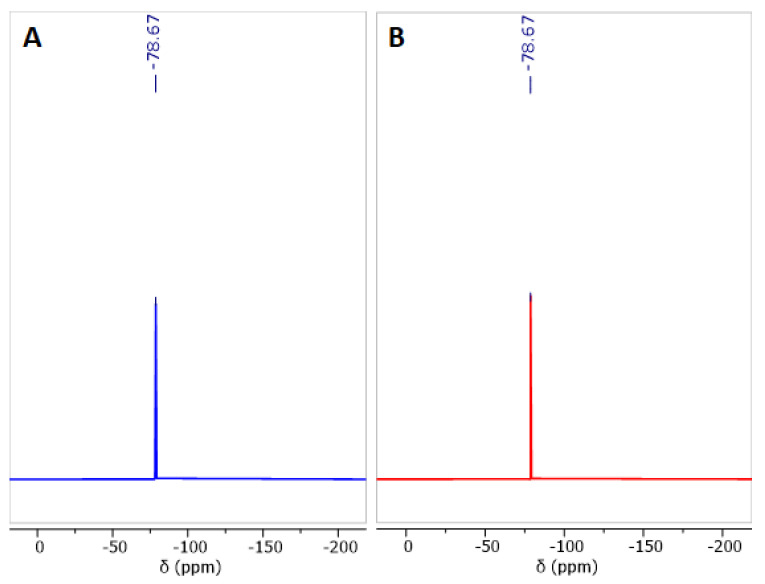
^19^F-NMR spectra of polyelectrolytes quaternized with methyl (**A**) and ethyl (**B**) groups. Measurements performed in DMSO-*d_6_*.

The apparition of carbon signals ascribed to TFSI^−^ counterions served as motivation to record ^19^F-NMR spectra, successfully demonstrating the presence of these anions in both polyelectrolytes. The results are shown below in Figure 8A,B.

So far, by complementing the results of FT-IR, ^1^H, ^13^C, and ^19^F-NMR measurements, we can confirm the correct chemical structure of both polyelectrolytes obtained from the alkylation of poly(ODA-co-PyMDA), demonstrating that the synthetic route designed and employed in this work is feasible. Table 2 summarizes the solubility test and viscosity measurements carried out for poly(ODA-co-PyMDA) and both polyelectrolytes.

The above table reveals a remarkably similar result in terms of solubility for all obtained samples, showing as the main difference the behavior exhibited by both polyelectrolytes against NMP. It is well-known that polyamides usually exhibit low solubility, due to the presence of strong intermolecular interactions such as hydrogen bonds and π–π stacking. However, all these polymers possess solubility in aprotic polar solvents frequently used in the manufacture and processability of polyamides. On the other hand, it draws attention to the abrupt increase of the viscosity when polyelectrolytes are compared against poly(ODA-co-PyMDA). The above phenomenon has been widely reported for charged polymers [38], where the higher viscosities shown by this type of macromolecules have been attributed to the tendency of their polymer chains to adopt more extended conformations in solution. This would be explained by the presence of electrostatic repulsions forces generated between monomeric units with the same charge. Therefore, considering that the molecular weight between the obtained materials is not changed, the increased viscosities values of poly(ODA-co-PyMDA[Me])TFSI^−^ and poly(ODA-co-PyMDA[Et])TFSI^−^ could be indicative of their charged nature.

### 3.1. Thermal Properties of Polymers

**Figure 9:**
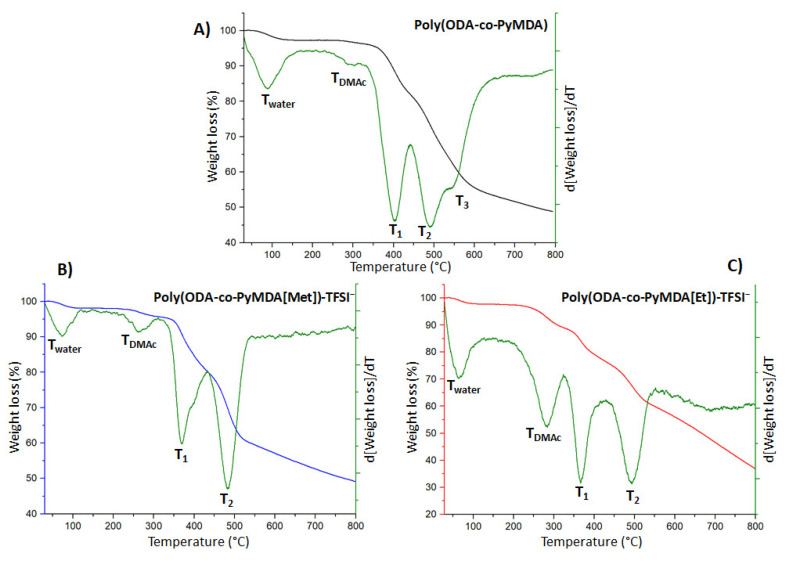
TGA thermograms obtained for poly(ODA-co-PyMDA) (**A**) and polyelectrolytes (**B**,**C**).

Once we achieved the structural characterization of the obtained polymers, their thermal properties were evaluated by means of thermogravimetric analysis (TGA) and differential scanning calorimetry (DSC). Figure 9A–C shows the thermal decomposition curves measured for poly(ODA-co-PyMDA) and both its polyelectrolytes, while Table 3 summarizes the main values regarding the thermal resistance of these polymers.

The discussion regarding the thermal resistance of the obtained polymers will start analyzing the thermal behavior of poly(ODA-co-PyMDA). Figure 9A shows a decomposition profile composed by five degradation steps, which were studied by direct comparison against thermograms obtained for poly(PyMDA) and poly(ODA) (Figure S4 and Table S2 Supporting Information), along with recording FT-IR spectra for samples obtained after each decomposition stage of poly(ODA-co-PyMDA), poly(ODA-co-PyMDA[Me])TFSI^−^, and poly(ODA-co-PyMDA[Et])TFSI^−^ (Figure S5). Thereby, steps labeled as T_water_ and T_DMAc_ were related to the volatilization of water and less volatile solvents such as DMAc, respectively, since no changes in the FT-IR spectra were observed before and after the thermal treatment up to these temperatures, ensuring a complete conservation of the polyamide chemical structure. Contrary to the above, degradations labeled as T_1_, T_2_, and T_3_ were accompanied with changes in the FT-IR of poly(ODA-co-PyMDA) and therefore are related with the degradation of certain structures of the polymer backbone. In this sense, it draws attention to how T_2_ degradation coincides well with the only decomposition observed for poly(ODA) and should be ascribed to the breaking and volatilization of fragments involving ODA moieties, while T_1_ and T_3_ appear at similar temperatures to those observed in the poly(PyMDA) thermogram and therefore would be related to the decomposition of structures containing PyMDA units. However, it is interesting to figure out the nature of T_1_ and T_3_ degradations. In order to achieve the above, theoretical calculations were performed to estimate the percentage weight contribution of pyridinyl pendant groups in the poly(PyMDA) structure. Calculations gave as a result that pyridinyl entities represent a 24.0% of the total mass, approaching quite well to the value 23.3% obtained from the first degradation step of poly(PyMDA). By repeating the same procedure, but now for poly(ODA-co-PyMDA), we obtain a value of 13.0% for the theoretical pyridinyl mass contribution, while the mass loss calculated from T_1_ was 12.2%, reinforcing the idea that T_1_ would be related to the rupture of side groups of PyMDA units. In order to confirm the above result, FT-IR analysis was performed to the obtained residue of poly(ODA-co-PyMDA) after being heated up to 440 °C (Figure S5). The spectrum reveals an intensity decrease of the amide carbonyl signal and the band related to C=N vibrations, which were both related to the pyridinyl pendant group. Finally, T_2_ and T_3_ would be related to the decomposition of ODA and PyMDA fragments forming part of the polymer main chain, respectively.

After the alkylation of poly(ODA-co-PyMDA), some relevant changes in the thermal degradation behavior were observed. Figure 9B,C expose TGA curves measured for both polyelectrolytes where, instead of five degradation steps, only four were detected and labeled as T_water_, T_DMAc_, T_1_, and T_2_. Similar to poly(ODA-co-PyMDA), the first two steps are attributed to the volatilization of low molecular weight species, which are mainly attributed to solvents. This was again supported by the recording of FT-IR spectra for both polyelectrolytes once they were heated up to 320 °C. It is worth noting that poly(ODA-co-PyMDA[Et])TFSI^−^ possesses a noticeable higher amount of retained solvent, which forced us to expose the three polymers to an extra drying process before continuing with the development of the present work. Regarding T_1_ in both polyelectrolytes, it can be seen that they occur at a lower temperature in comparison to poly(ODA-co-PyMDA), which would be indicative of pyridinyl groups with a more labile structure once its alkylation is achieved. Finally, the decomposition labeled as T_2_ in both polyelectrolytes could arise from the merging of the two steps previously labeled as T_2_ and T_3_ in poly(ODA-co-PyMDA) and therefore would be related to the decomposition of the polymer backbone. This merging could be originated since, apparently, both degradation steps associated with PyMDA structures shift to lower temperatures.

**Figure 10:**
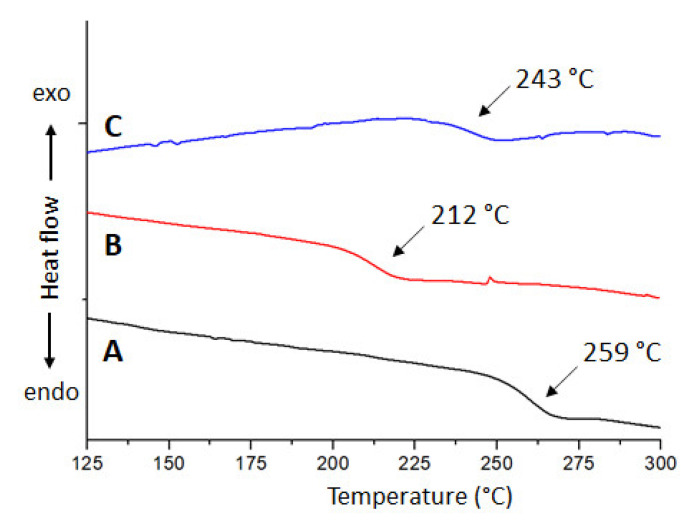
DSC curves of poly(ODA-co-PyMDA) (**A**), poly(ODA-co-PyMDA[Me])TFSI^−^, (**B**) and poly(ODA-co-PyMDA[Et])TFSI^−^ (**C**).

To end the thermal characterization, Figure 10A–C exhibits DSC measurements carried out for these materials, where all of them showed an amorphous behavior with their glass transition temperatures (T_g_) indicated by black arrows. We must point out the lowest T_g_ values exhibited by both polyelectrolytes in comparison with the neutral polyamide. This could be explained in terms of a lower packing degree of chains after the pyridinyl groups’ alkylation, which originated by charge repulsion forces and the steric hindrance of TFSI counterions.

Based on the previously exposed, all obtained polymer exhibit degradation onset temperatures above 300 °C and T_g_ values well above the boiling point of water, allowing them to be considered as polymers with outstanding thermal properties for water treatment applications.

### 3.2. Mechanical Properties of Polymers

**Figure 11:**
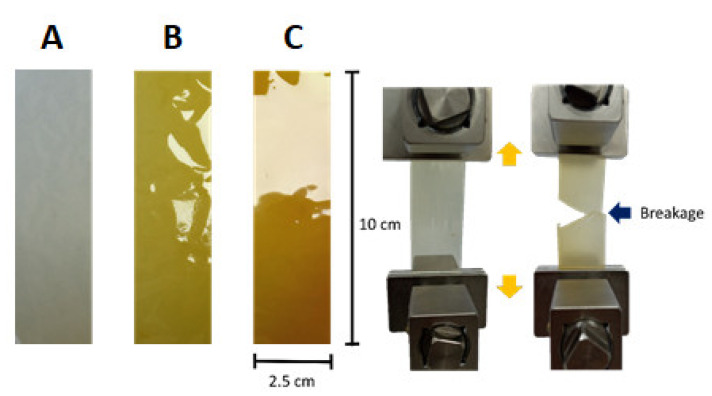
Films obtained from poly(ODA-co-PyMDA) (**A**), poly(ODA-co-PyMDA[Me])TFSI^−^, (**B**) and poly(ODA-co-PyMDA[Et])TFSI^−^ (**C**). To the right, a representation of the tensile test performed.

Continuing with the discussion, poly(ODA-co-PyMDA) and its both polyelectrolytes were successfully processed in the form of flexible films, as is shown in Figure 11A–C, and they were subjected to tensile tests in order to evaluate their mechanical properties, which are summarized in Table 4.

The results exposed in Table 4 reveal that the mechanical properties of poly(ODA-co-PyMDA) are not greatly affected after its transformation into polyelectrolytes. However, looking for some tendencies, it can be seen that both polyelectrolytes exhibited lower Young’s modulus values and also a diminishing of their yield strength values. Thereby, as a general result, polyelectrolytes seem to acquire a greater elasticity supported by the increase of tensile strength and elongation at break values. These charged polymers exhibit a higher flexibility and resistance against deformation in comparison to the non-charged polyamide; a feasible explanation to the above could be related to the higher free volume existing between their chains due to the presence of bulky counterions and electrostatic repulsion forces. This greater free volume, also supported by DSC results, can be visualized as an increased distance between chains and, therefore, lower interchain interactions, which under mechanical stress allow the displacement of chains and their re-accommodation before inducing the rupture of the material.

### 3.3. Hydrophilic and Hydrophobic Properties of Polymers

**Figure 12:**
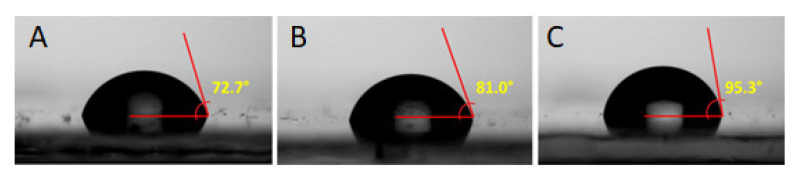
Water contact angle measurements performed onto poly(ODA-co-PyMDA) (**A**), poly(ODA-co-PyMDA[Me])TFSI^−^ (**B**), and poly(ODA-co-PyMDA[Et])TFSI^−^ (**C**) films.

Approaching the study of these materials in water desalination applications, it is essential to examine the behavior of the obtained films against water. To afford this, in a first stage, wettability studies were carried out by contact angle measurements. Figure 12A–C show images of measurements carried out, whose values are summarized in Table 5.

At first sight, it draws attention the higher values exhibited by both polyelectrolytes in comparison to poly(ODA-co-PyMDA), revealing a greater hydrophobic balance from the charged polymers. Considering the normal behavior of classical polyelectrolytes, the above result was not expected, since the presence of charges along the polymer backbone should increase the interaction with polar molecules, such as water. However, for this particular case, the importance of the counterion used must be pointed out. In recent years, due to the development of poly(ionic liquids) (PILs), the TFSI molecule, along with many others, has been tested as counterions of polycationic species. Particularly, and very related to this work, Briones et al. [39] have synthetized and evaluated the properties of novel poly(ionic liquids) prepared from quaternized poly(4-vinylpyridine) and different counterions, TFSI being one of them. Based on the above, we can stipulate that alkylated PyMDA moieties containing TFSI counterions could be considered as an ionic liquid-like structure, and therefore, the polyelectrolytes obtained in this work could be considered as poly(ionic liquids). Notwithstanding the above, at the moment, the authors of the present work prefer to refer to these polymers as polyelectrolytes since, before being classified as poly(ionic liquids), we believe that further and deeper studies must be carried out in order to evaluate their properties. However, it must be pointed out that a great number of PILs have shown a marked hydrophobic character. As an example, Welton et al. [40] have successfully quantified the hydrophobicity of a number of imidazolium-based PILs, some of them containing TFSI counterions, by measuring their octanol–water partition coefficients. Having said the above, the higher values of water contact angles obtained for both polyelectrolytes could be ascribed to the presence of these TFSI counterions. However, in addition to the above, it could be possible that the presence of charged species inside of the polyamide matrix could generate a certain degree of interactions with other polar structures (e.g., amide functionalities), affecting their availability to interact with other substances.

**Figure 13:**
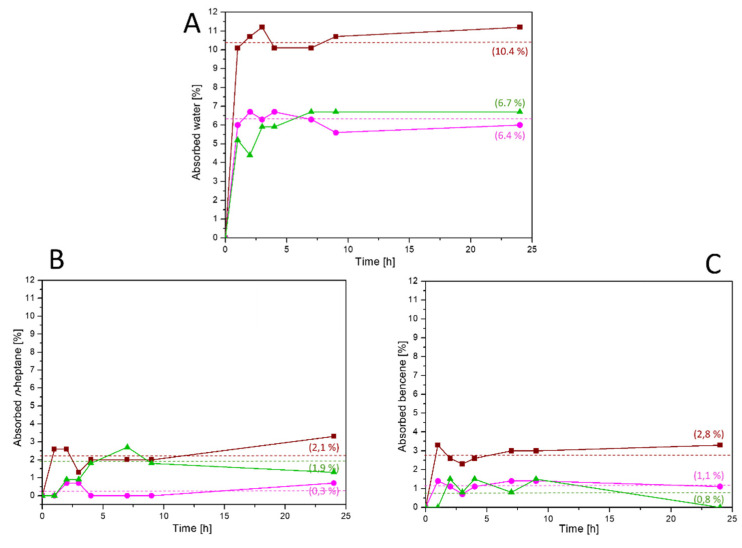
Water (**A**), *n*-heptane (**B**), and benzene (**C**) absorption curves recorded for poly(ODA-co-PyMDA) (wine), poly(ODA-co-PyMDA[Me])TFSI^−^ (magenta), and poly(ODA-co-PyMDA[Et])TFSI^−^ (green) films.

The higher hydrophobic character exhibited by these polyelectrolytes against poly(ODA-co-PyMDA) indicates the importance of the alkylation and subsequent counterion replacement processes, since for water desalination applications, it is mandatory to avoid, as much is possible, the solubilization and/or degradation of the material by the action of water. On the other hand, it is also important to diminish the membrane’s contamination by the action of organic species always present in residual waters. Thereby, contact angle measurements were carried out using n-heptane as an organic contaminant model, showing greater repellency to this substance by both polyelectrolytes (see the Supporting Information in Figure S6). Regarding the above, it seems that the charged nature of these materials provided by the alkylation of PyMDA units, along with the presence of TFSI counterions, promotes a lower affinity to both polar and apolar substances. However, in order to evaluate this behavior under a more “real” scenario, absorptions test were carried out by immersing films of poly(ODA-co-PyMDA) and both polyelectrolytes films in water, *n*-heptane, and benzene. Benzene was included in this study as a model of aromatic apolar substances, which are also detected in residual waters. The absorption test results are shown in Figure 13A–C.

The absorption tests correlate quite well with the results obtained from contact angle measurements, confirming the tendency of both polyelectrolytes to exhibit a lower affinity against polar and non-polar substances, which are represented by water, *n*-heptane, and benzene. Thereby, the preparation of polyelectrolytes bearing ionic liquid-like counterions from poly(ODA-co-PyMDA) is effective in creating new polyamide-based materials for use in water treatment applications. This is a result of high relevance, since it could mean that the inner structure of both polyelectrolytes should be less affected during contact with residual waters. Therefore, as was mentioned above, the hydrophobic character provided by the nature of TFSI anions should explain the lower water retention, while the same charged nature of materials would induce a decrease in the affinity toward non-polar substances, allowing stating that TFSI moieties could fulfill a dual function.

### 3.4. Preliminary Evaluation of Membrane Performance

**Figure 14:**
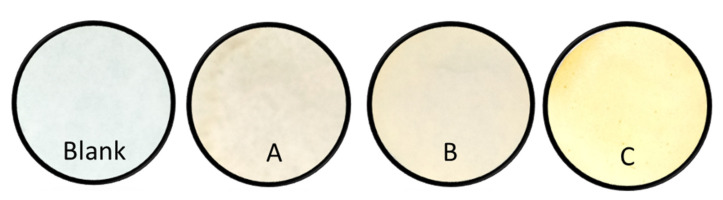
Membranes (filter disc coated with poly(ODA-co-PyMDA) (**A**), poly(ODA-co-PyMDA[Me])TFSI^−^ (**B**), and poly(ODA-co-PyMDA[Et])TFSI^−^ (**C**)).

With the aim of testing these materials in water desalination treatments, a preliminary evaluation was carried out on the membranes manufactured from a filtration disk coated with a thin layer of poly(ODA-co-PyMDA) and both polyelectrolytes, whose thicknesses ranged from 11.4 to 17.1 µm (Figure 14A–C). The results shown in Table 6 indicate that the flux decreases notoriously for the membranes manufactured with poly(ODA-co-PyMDA[Me])TFSI^−^ and poly(ODA-co-PyMDA[Et])TFSI^−^, being much lower for poly(ODA-co-PyMDA), which is derived from the increase in thickness of the membranes. This effect was also observed in the rejection of the membranes to the passage of NaCl, which was also consistent with their higher hydrophobicity.

On the other hand, when the results obtained at 1000 and 2000 ppm are compared, it is observed that all the membranes had a better performance with a lower concentration, both in flow and rejection. Regarding the lower flux compared to higher concentrations of salt, it is believed that it comes from the greater difficulty faced by the ions during their diffusion through the membrane, while the lower rejection indicates a saturation of the membrane during the passage of the ions, so that after reaching this condition, the membranes allow the free passage of these.

Although the preliminary tests showed the feasibility of using these materials for the manufacture of membranes applicable in water treatment, there are still several important aspects that must be evaluated, such as knowing and controlling the porosity, roughness, and uniformity of the membranes. These are aspects that will be developed and studied in the next stage of the research, where an optimization will be carried out in the manufacture of the membranes from the synthesized polymers.

## 4. Conclusions

From the results obtained, it can be concluded that it is possible to obtain a soluble and high molecular weight aromatic copolyamide that presents pendant pyridinyl groups easily alkylated with short-chain alkyl iodides such as ethyl and methyl, giving rise to a new group of polyelectrolytes that has similar characteristics to the original polymer such as high molecular weight, good solubility in aprotic polar solvents, high thermal stability (over 300 °C), and moderate mechanical resistance, all of which is derived from the synergy between the pyridinyl pendant groups and oxyether, aromatic, and amide groups present in the chain. In addition, these materials have a low interaction with both polar and apolar substances, product of the quaternization of the pendant group and the counterion used, making them attractive for the manufacture of membranes for water treatment.

## Figures and Tables

**Table 1:** Number-average molecular weight (Mn), weight-average molecular weight (M_w_), and dispersity (Đ) values calculated for poly(ODA-co-PyMDA).

	M_n_ (g/mol)	M_w_ (g/mol)	Đ
poly(ODA-co-PyMDA)	56,290	96,060	1.71

**Table 2:** Solubility and viscosity values of obtained polymers.

Solvents	Polymers ^a^		
	Poly(ODA-co-PyMDA)	Poly(ODA-co-PyMDA[Me])TFSI^−^	Poly(ODA-co-PyMDA[Et])TFSI^−^
H_2_O	-	-	-
EtOH	-	-	-
MeOH	-	-	-
*n*-hexane	-	-	-
Acetone	-	-	-
CHCl_3_	-	-	-
THF	-	-	-
AcOEt	-	-	-
1,2-Dioxane	-	-	-
DMF	+	+	+
DMA	+	+	+
NMP	+	-	-
DMSO	+	+	+
*η*_inh_ (dL/g) ^b^	0.80	1.19	2.38

^a^ c = 10 mg in 0.5 mL of solvent. + soluble at RT, +/- soluble at 40 °C, -insoluble. ^b^ [C] = 50 mg in 10 mL of DMSO at 30 °C.

**Table 3:** Thermal properties of obtained polymers.

Polymer	T_i_ (°C) ^a^	T_1_ (°C)	T_2_ (°C)	T_3_ (°C)	R (%) ^b^
**poly(ODA-co-PyMDA)**	395	402	492	544	49
**poly(ODA-co-PyMDA[Me])TFSI^−^**	373	370	483	-	49
**poly(ODA-co-PyMDA[Et])TFSI^−^**	309	367	492	-	37

^a^ Degradation onset temperature measured as the temperature at which the material loses 10% of its initial mass; ^b^ Solid residues generated after pyrolysis.

**Table 4:** Mechanical properties of obtained polymers.

Polymer	Young’s Modulus	Yield Strength	Tensile Strength	Elongation at Break
	[Gpa]	[Mpa]	[Mpa]	[%]
poly(ODA-co-PyMDA)	2.7 ± 0.03	56.3 ± 1.5	76.7 ± 1.4	4.9 ± 0.5
poly(ODA-co-PyMDA[Me])TFSI^−^	2.4 ± 0.02	48.4 ± 1.1	87.9 ± 1.2	7.5 ± 0.5
poly(ODA-co-PyMDA[Et])TFSI^−^	2.5 ± 0.04	44.5 ± 0.8	83.5 ± 1.6	5.6 ± 0.3

**Table 5:** Contact angle values measured for obtained films.

Polymer	Water Contact Angle
poly(ODA-co-PyMDA)	72.7 ± 2.1
poly(ODA-co-PyMDA[Me])TFSI^−^	81.0 ± 2.1
poly(ODA-co-PyMDA[Et])TFSI^−^	95.3 ± 2.4

**Table 6:** Membrane performance evaluation results.

Sample	Thickness [µm]	Time [s]	Flux [L/m^2^h]	Concentration [mg/L]	Rejection [%]
**Blank 1000 ^a^**	0	6.76	55,379.6	979	2.1
**Blank 2000 ^a^**	0	7.67	48,809.2	1988	0.6
**A 1000**	11.4 ± 1.0	10.83	34,567.5	909	9.1
**A 2000**	11.4 ± 1.0	8.98	41,688.9	1893	5.4
**B 1000**	12.6 ± 1.5	46.60	9220.8	861	13.9
**B 2000**	12.6 ± 1.5	108.00	3466.4	1875	6.2
**C 1000 ^b^**	17.1 ± 0.5	12.6 × 10^3^	29.7	797	20.3
**C 2000 ^b^**	17.1 ± 0.5	14.4 × 10^3^	26.0	1826	8.7

**Conditions:** solution volume 100 mL, vacuum pressure 14 cm Hg, temperature 25 °C. (^a^) only filter disc. (^b^) vacuum pressure **=** 40 cm Hg.

## Data Availability

Data is contained within the article or Supplementary Material.

## Supplementary Materials

The following are available online at https://www.mdpi.com/article/10.3390/polym13121993/s1, Image S1: Experimental assembly for stage one in the synthesis of 3,5-dinitro-*N*-(pyridin-4-ylmethyl)benzamide (PyMDN), addition of 3,5-dinitrobenzoyl chloride upon dissolution of pyridin-4-ylmethanoamine (A) and experimental assembly for stage two in the synthesis of PyMDN, heating-agitation and synthesis PyMDA by reduction of PyMDN with Pd/C and hydrazine (B), Image S2: Experimental assembly for the synthesis of polymers (A) and polyelectrolyte (B), Scheme S1: Chemical structure of poly(PyMDA), Scheme S2: Chemical structure of poly(ODA), Scheme S3: Chemical structure of Poly(ODA-co-PyMDA[Bu])-I^−^, Scheme S4: Chemical structure of Poly(ODA-co-PyMDA[Hex])-I^−^), Figure S1: FT-IR spectra of poly(PyMDA) (A), poly(PyMDA-co-ODA) (B) and poly(ODA) (C), Figure S2: ^1^H NMR spectra of poly(PyMDA) (A), poly(PyMDA-co-ODA) (B) and poly(ODA) (C) (400 MHz, DMSO-*d_6_*), Figure S3: ^13^C NMR spectra of poly(PyMDA) (A), poly(PyMDA-co-ODA) (B) and poly(ODA) (C) (400 MHz, DMSO-*d_6_*), Figure S4: TGA (A) and DTGA (B) thermograms of poly(PyMDA), poly(PyMDA-co-ODA) and poly(ODA), Figure S5. Comparison of FT-IR spectra of poly(ODA-co-PyMDA) (A), poly (ODA-co-PyMDA [Me]) TFSI^−^ (B) and poly (ODA-co-PyMDA [Et]) TFSI^−^ (C) with its waste (poly(ODA-co-PyMDA) waste (Aw), poly(ODA-co-PyMDA [Me])TFSI^−^ waste, (Bw) and poly(ODA-co-PyMDA [Et])TFSI^-^ waste (Cw)) after heating to 420 and 320 °C, Figure S6. Images of n-heptane drop on poly(ODA-co-PyMDA) (A), poly (ODA-co-PyMDA [Me]) TFSI^−^ (B) and poly (ODA-co-PyMDA [Et]) TFSI^−^ (C), Table S1: Solubility and viscosity values of polyamides, Table S2. Polymers thermal properties.

## References

[1] Avtar R., Tripathi S., Aggarwal A.K., Kumar P. (2019). Population–Urbanization–Energy Nexus: A Review. Resources.

[2] Gupta R., Yan K., Singh T., Mo D. (2020). Domestic and International Drivers of the Demand for Water Resources in the Context of Water Scarcity: A Cross-Country Study. J. Risk Financ. Manag..

[3] Tripathi A.D., Mishra R., Maurya K.K., Singh R.B., Wilson D.W. (2019). Estimates for World Population and Global Food Availability for Global Health. Role Funct. Food Secur. Glob. Health.

[4] Jorquera H., Montoya L.D., Rojas N.Y. (2019). Chapter 7-Urban Air Pollution. Urban. Clim. Latin Am..

[5] Dolan F., Lamontagne J., Link R., Hejazi M., Reed P., Edmonds J. (2021). Evaluating the economic impact of water scarcity in a changing world. Nat. Commun..

[6] Falkenmark M. (2020). Water resilience and human life support—Global outlook for the next half century. Int. J. Water Resour. Dev..

[7] Ezugbe E.O., Rathilal S. (2020). Membrane Technologies in Wastewater Treatment: A Review. Membranes.

[8] Quist-Jensen C., Macedonio F., Drioli E. (2015). Membrane technology for water production in agriculture: Desalination and wastewater reuse. Desalination.

[9] Ravanchi M.T., Kaghazchi T., Kargari A. (2009). Application of membrane separation processes in petrochemical industry: A review. Desalination.

[10] Doan H., Lohi A. (2013). Fouling in Membrane Filtration and Remediation Methods. Mass Transf. Adv. Sustain. Energy Environ. Oriented Numer. Modeling.

[11] Lau W.J., Ismail A.F., Misdan N., Kassim M.A. (2012). A recent progress in thin film composite membrane: A review. Desalination.

[12] Nataraj S., Hosamani K., Aminabhavi T. (2009). Nanofiltration and reverse osmosis thin film composite membrane module for the removal of dye and salts from the simulated mixtures. Desalination.

[13] Mulder M. (2000). Chapter 3-MEMBRANE PREPARATION | Phase Inversion Membranes. Encycl. Sep. Sci..

[14] Kim K., Lee K., Cho K., Park C. (2002). Surface modification of polysulfone ultrafiltration membrane by oxygen plasma treatment. J. Membr. Sci..

[15] Liu M., Wu D., Yu S., Gao C. (2009). Influence of the polyacyl chloride structure on the reverse osmosis performance, surface properties and chlorine stability of the thin-film composite polyamide membranes. J. Membr. Sci..

[16] Cadotte J.E. (1981). Interfacially Synthesized Reverse Osmosis Membrane. U.S. Patent.

[17] Garcia J., García F.C., Serna F., De La Peña J.L. (2010). High-performance aromatic polyamides. Prog. Polym. Sci..

[18] Kwak S.-Y., Jung S.G., Kim S.H. (2001). Structure-Motion-Performance Relationship of Flux-Enhanced Reverse Osmosis (RO) Membranes Composed of Aromatic Polyamide Thin Films. Environ. Sci. Technol..

[19] Agenson K.O., Urase T. (2007). Change in membrane performance due to organic fouling in nanofiltration (NF)/reverse osmosis (RO) applications. Sep. Purif. Technol..

[20] Shintani T., Matsuyama H., Kurata N. (2007). Development of a chlorine-resistant polyamide reverse osmosis membrane. Desalination.

[21] Hirose M., Ito H., Kamiyama Y. (1996). Effect of skin layer surface structures on the flux behaviour of RO membranes. J. Membr. Sci..

[22] Al-Jeshi S., Neville A. (2006). An investigation into the relationship between flux and roughness on RO membranes using scanning probe microscopy. Desalination.

[23] Ruiz J.A.R., Trigo-López M., García F.C., García J.M. (2017). Functional Aromatic Polyamides. Polymer.

[24] Espeso J.G., de la campa A.G., Lozano A.G., de abajo J. (2000). Synthesis and Characterization of New Soluble Aromatic Polyamides Based on 4-(1-Adamantyl)-1,3-bis(4-aminophenoxy)benzene. J. Polym. Sci. Part. A Polym. Chem..

[25] Hsiao S.-H., Chen C.-W., Liou G.-S. (2004). Novel aromatic polyamides bearing pendent diphenylamino or carbazolyl groups. J. Polym. Sci. Part. A Polym. Chem..

[26] Terraza C.A., Tagle L.H., Tundidor-Camba A., González-Henríquez C., Ortiz P., Coll D. (2012). Poly(amide)s obtained from 4-(4-((4-(4-aminophenoxy)phenyl)diphenylsilyl)phenoxy)benzenamine and dicarboxylic acids containing diphenylsilarylene units. Synth. Charact. Eur. Polym. J..

[27] Ye L., Wang L., Jie X., Yu C., Kang G., Cao Y. (2020). Effect of hexafluoroisopropylidene group contents and treatment temperature on the performance of thermally rearranged poly(hydroxyamide)s membranes. J. Membr. Sci..

[28] Patil A., Medhi M., Sadavarte N., Wadgaonkar P., Maldar N. (2010). Synthesis and characterization of novel aromatic–aliphatic polyamides from bis-[(4-aminobenzyl)-4-benzamide] ether. Mater. Sci. Eng. B.

[29] Li X., Liu C., Van der Bruggen B. (2020). Polyelectrolytes self-assembly: Versatile membrane fabrication strategy. J. Mater. Chem. A.

[30] Singh P.K., Singh V.K., Singh M. (2007). Zwitterionic Polyelectrolytes: A Review. e-Polymers.

[31] Kim G.-T., Appetecchi G.B., Alessandrini F., Passerini S. (2007). Solvent-free, PYR1ATFSI ionic liquid-based ternary polymer electrolyte systems: I. Electrochemical characterization. J. Power Sources.

[32] Shaplov A.S., Ponkratov D.O., Vygodskii Y.S. (2016). Poly(ionic liquid)s: Synthesis, properties, and application. Polym. Sci. Ser. B.

[33] Rathnayake R.M.L.L., Perera K.S., Vidanapathirana K.P. (2020). Past, present and future of ionic liquid based polymer electrolytes. AIMS Energy.

[34] Bara J.E., O’Harra K.E. (2019). Recent Advances in the Design of Ionenes: Toward Convergence with High-Performance Polymers. Macromol. Chem. Phys..

[35] Yuan J., Antonietti M. (2015). Poly(Ionic Liquid)s as Ionic Liquid-Based Innovative Polyelectrolytes. Appl. Ion. Liq. Polym. Sci. Technol..

[36] Tundidor-Camba A., Saldias C., Tagle L.H., Terraza C.A., Coll D., Pérez G., Aguilar-Vega M., Abarca R.L., Ortiz P.A. (2018). Synthesis, characterization and film preparation of new co-polyimide based on new 3,5-diamino-N-(pyridin-4-ylmethyl)benzamide, ODA and 6FDA. J. Chil. Chem. Soc..

[37] Yamazaki N., Matsumoto M., Higashi F. (1975). Studies on reactions of the N-phosphonium salts of pyridines. XIV. Wholly aromatic polyamides by the direct polycondensation reaction by using phosphites in the presence of metal salts. J. Polym. Sci. Polym. Chem. Ed..

[38] Fuoss R.M., MacLay W.N. (1951). Polyelectrolytes. VI. Viscosities of 4-polyvinylpyridine hydrochloride in methanol at 25°. J. Polym. Sci..

[39] Briones X., Tapia R.A., Campodónico P.R., Urzúa M., Leiva A., Contreras R., González-Navarrete J. (2018). Synthesis and characterization of poly (ionic liquid) derivatives of N-alkyl quaternized poly(4-vinylpyridine). React. Funct. Polym..

[40] Hallett J., Welton T. (2011). Room-Temperature Ionic Liquids: Solvents for Synthesis and Catalysis. 2. Chem. Rev..

