# Supplementary material for: Novel Polyelectrolytes Obtained by Direct Alkylation and Ion Replacement of a New Aromatic Polyamide Copolymer Bearing Pyridinyl Pendant Groups
Source: Polymers (Basel). 2021 Jun 18;13(12):1993. doi: 10.3390/polym13121993 (PMC8233918; doi:10.3390/polym13121993)
Supplement: Supplementary file 1 [file polymers-13-01993-s001.zip › polymers-1261218-supplementary.pdf]

Supplementary Materials

Novel polyelectrolytes obtained by direct alkylation and ion-replacement of a new aromatic polyamide copolymer bearing pyridinyl pendant groups.

Sebastián Bonardd, Alejandro Ángel, Ángel Norambuena, Deysma Coll, Alain Tundidor-Camba, and Pablo A Ortiz.

Synthetic equipment setup

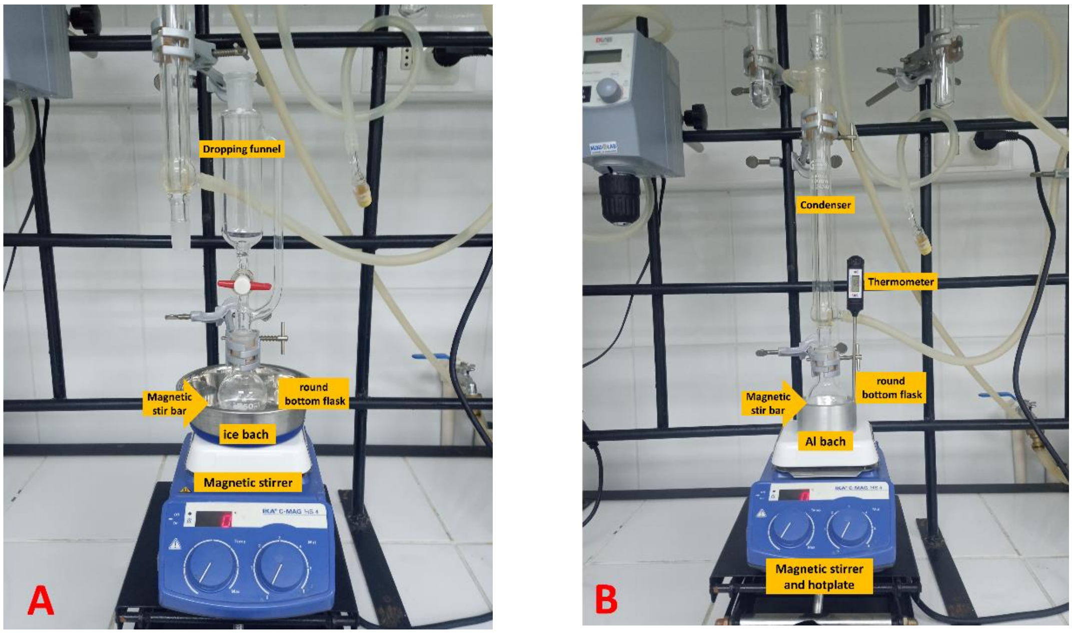

Image S1. Experimental assembly for stage one in the synthesis of 3,5-dinitro-*N*-(pyridin-4-ylmethyl)benzamide (PyMDN), addition of 3,5-dinitrobenzoyl chloride upon dissolution of pyridin-4-ylmethanoamine (A) and experimental assembly for stage two in the synthesis of PyMDN, heating-agitation and synthesis PyMDA by reduction of PyMDN with Pd/C and hydrazine (B).

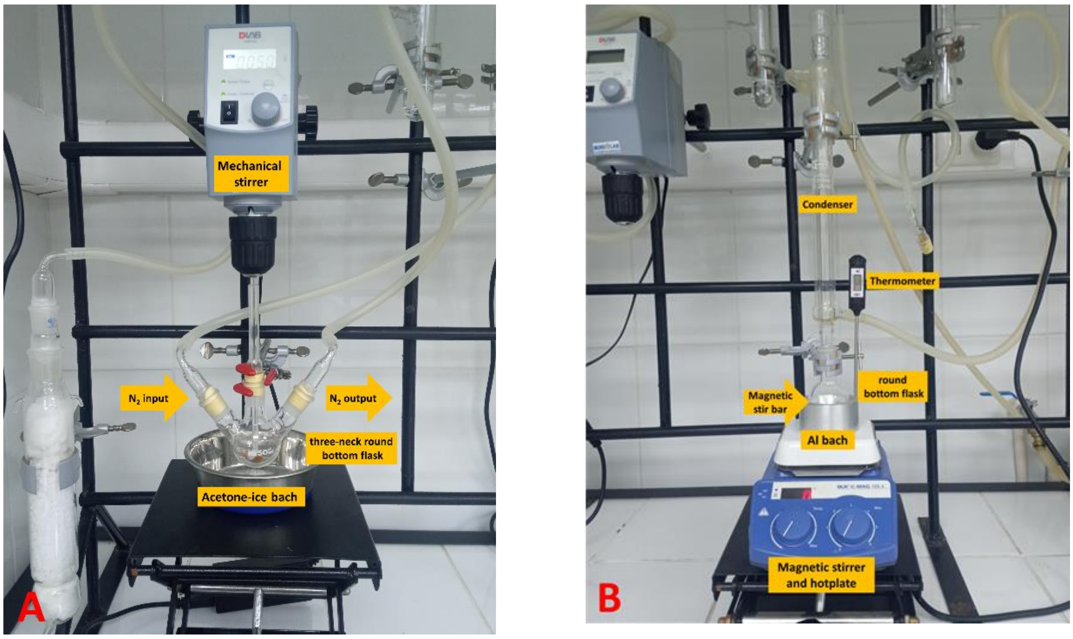

Image S2. Experimental assembly for the synthesis of polymers (A) and polyelectrolyte (B).

Spectroscopic data of polymers

Poly(ODA-co-PyMDA[Bu])-I . Yield: 99 %. FT-IR-ATR (ZnSe, cm^{-1}): 3231 (N-H); 3025 (C-H arom.); 1647 (C=O); 1602, 1445 (C=C); 1534 (C=N); 1496 (N-H); 1233, 1213 (C-N); 770, 720 (mono-subst.). There are no NMR spectra due to the insolubility of the polymer obtained.

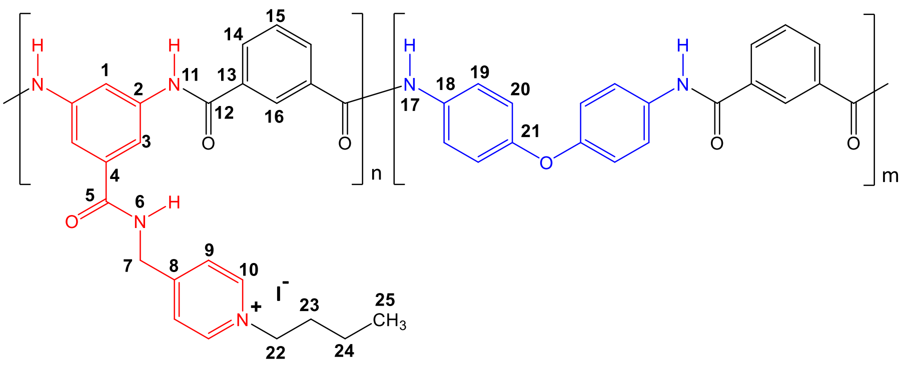

Scheme S1. Chemical structure of poly(ODA-co-PyMDA[Bu])-I^-.

Poly(ODA-co-PyMDA[Hex])-I . Yield: 99 %. FT-IR-ATR (ZnSe, cm^{-1}): 3231 (N-H); 3025 (C-H arom.); 1647 (C=O); 1602, 1445 (C=C); 1534 (C=N); 1496 (N-H); 1233, 1213 (C-N); 770, 720 (mono-subst.). There are no NMR spectra due to the insolubility of the polymer obtained.

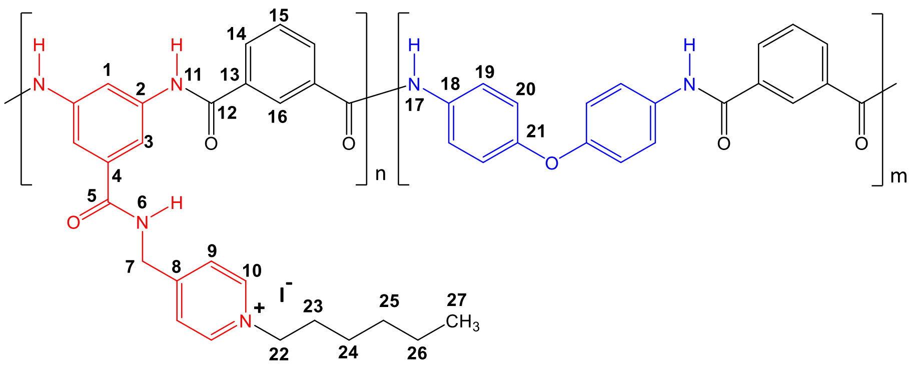

Scheme S2. Chemical structure of poly(ODA-co-PyMDA[Hex])-I^-).

Poly(PyMDA). Yield: 71 %. FT-IR-ATR (ZnSe, cm^{-1}): 3232 (N-H); 3068 (C-H arom.); 1654 (C=O); 1594, 1440 (C=C); 1534 (C=N); 1263, 1236 (C-N); 864, 675 (1,3-disubst); 765, 713 (mono-subst.). ^{1}H NMR (DMSO-d_6, δ, ppm): 10.70 (s, 2H, 11); 9.18 (t, 1H, 6); 8.63 (s, 1H, 16); 8.54 (d, 2H, 10); 8.20 (d, 2H, 14); 8.06 (s, 2H, 3); 7.99 (s, 1H, 1); 7.73 (t, 1H, 15); 7.37 (d, 2H, 9); 4.51 (d, 2H, 7). ^{13}C NMR (DMSO-d_6, δ, ppm): 166.8 (5); 165.3 (12); 149.0 (10); 139.4 (2); 135.7 (8); 135.4 (4); 135.0 (13); 130.9 (14); 128.8 (15); 127.3 (16); 122.4 (9); 115.8 (1); 115.4 (3); 41.6 (7).

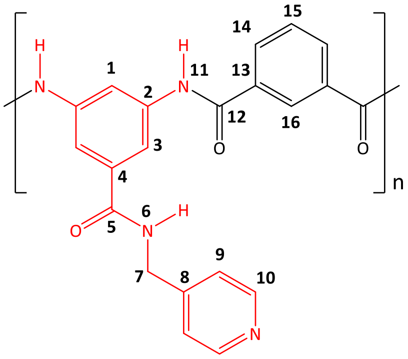

Scheme S3. Chemical structure of poly(PyMDA).

Poly(ODA). Yield: 82 %. FT-IR-ATR (ZnSe, cm^{-1}): 3268 (N-H); 3052 (C-H arom.); 1648 (C=O); 1600, 1494 (C=C); 1534 (C=N); 1216 (C-N); 828 (1,4-disubst); 878, 695 (1,3-disubst). ^{1}H NMR (DMSO-d_6, δ, ppm): 10.47 (s, 1H, 6); 8.56 (s, 1H, 5); 8.15 (d, 2H, 3); 7.82 (d, 4H, 8); 7.70 (t, 1H, 4); 7.05 (d, 4H, 9). ^{13}C NMR (DMSO-d_6, δ, ppm): 164.9 (1); 153.0 (10); 135.1 (2); 134.6 (7); 130.6 (3); 128.6 (4); 126.9 (5); 122.1 (8); 118.7 (9); 41.6 (7).

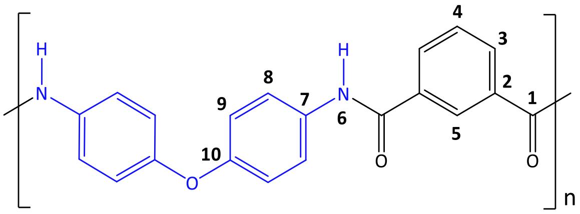

Scheme S4. Chemical structure of poly(ODA).

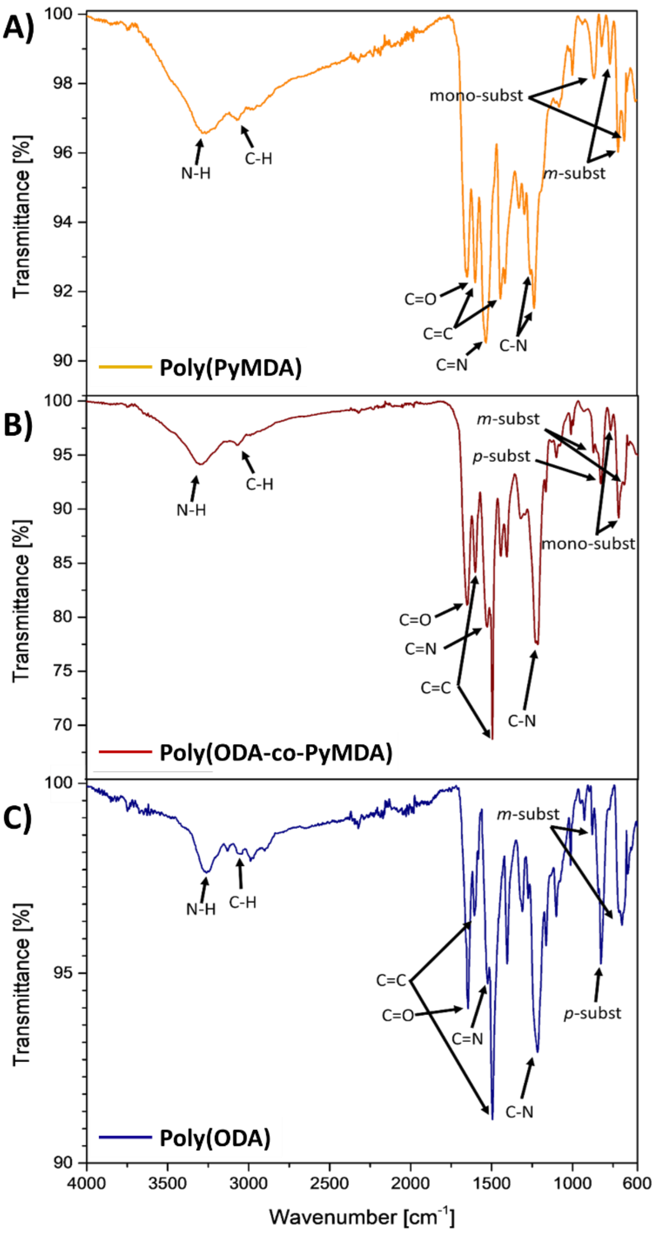

Figure S1. FT-IR spectra of poly(PyMDA) (A), poly(PyMDA-co-ODA) (B) and poly(ODA) (C).

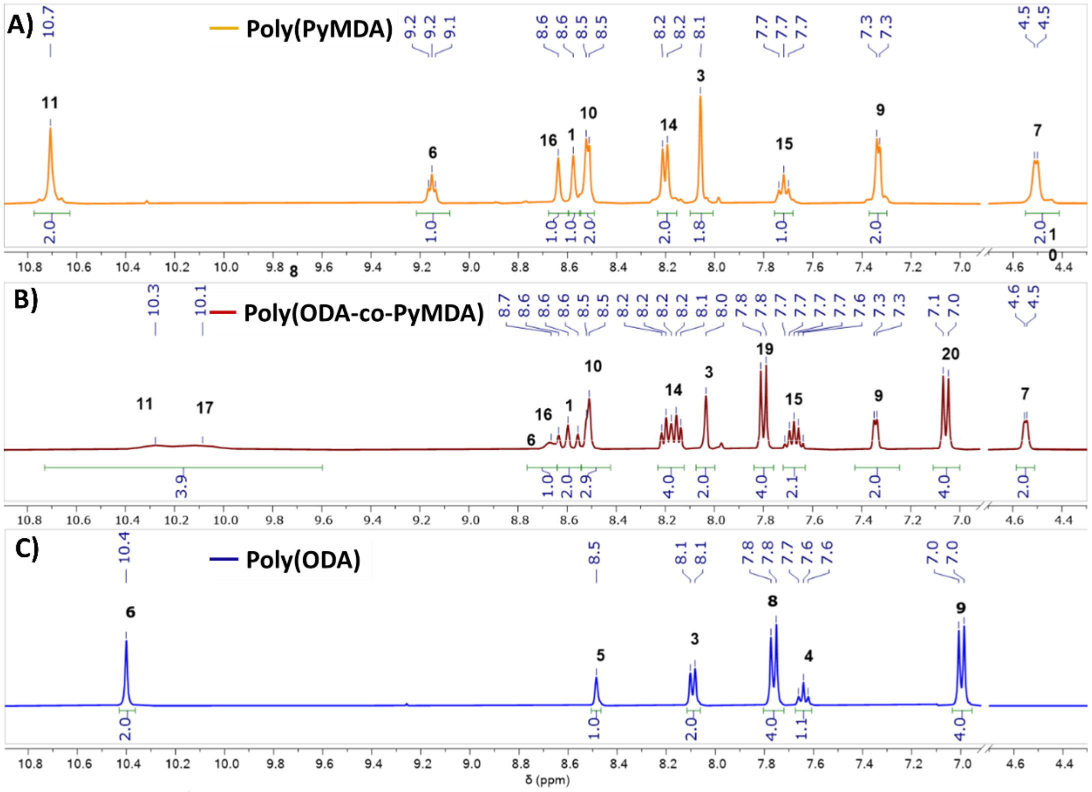

Figure S2. ^{1}H NMR spectra of poly(PyMDA) (A), poly(PyMDA-co-ODA) (B) and poly(ODA) (C) (400 MHz, DMSO-d_6).

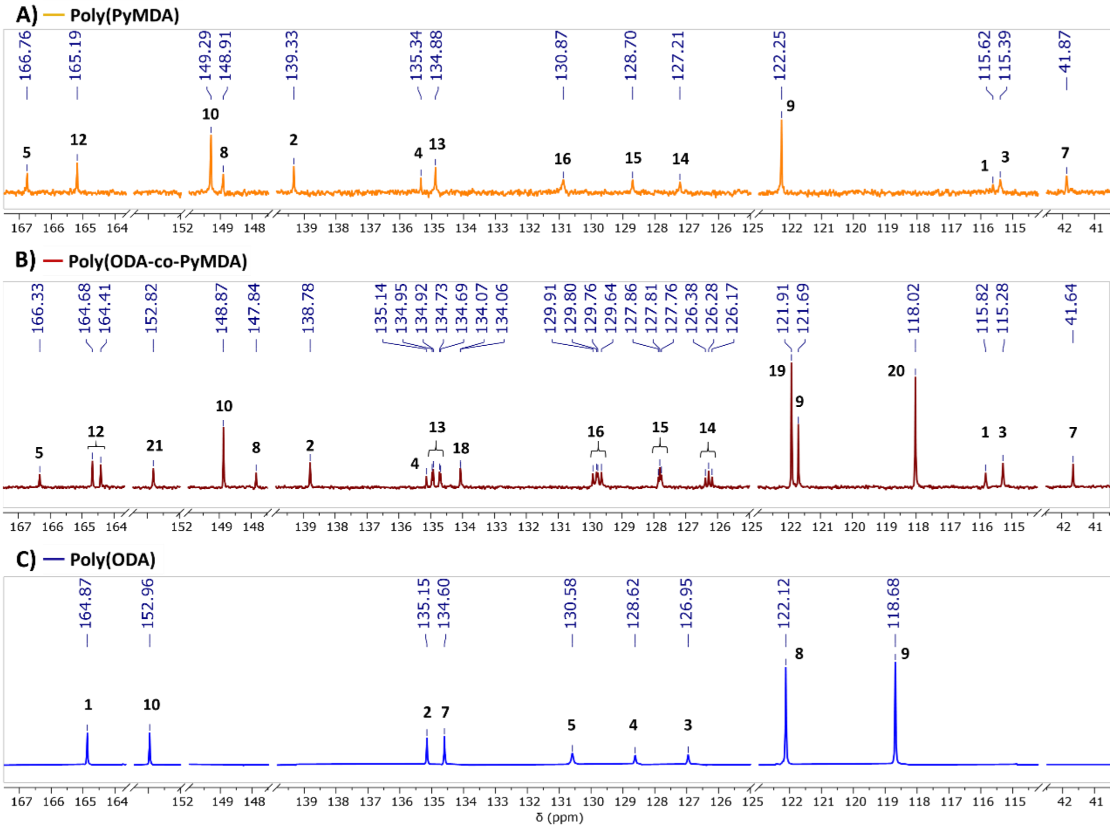

Figure S3. ^{13}C NMR spectra of poly(PyMDA) (A), poly(PyMDA-co-ODA) (B) and poly(ODA) (C) (400 MHz, DMSO-d_6).

Solubility and viscosity of polymers

Table S1. Solubility and viscosity values of polyamides

**Solvents**	**Polymers**[a]		
	poly(PyMDA)	poly(PyMDA-co-ODA)	poly(ODA)
**H_2O**	-	-	-
**EtOH**	-	-	-
**MeOH**	-	-	-
***n*-hexane**	-	-	-
**Acetone**	-	-	-
**$CHCl_3$**	-	-	-
**THF**	-	-	-
**AcOEt**	-	-	-
**1,2-Dioxane**	-	-	-
**DMF**	+	+	+
**DMA**	+	+	+
**NMP**	+/-	+	+
**DMSO**	+	+	+
**η_{inh} (dL/g)**[b]	0.36	0.80	0.77

[a] c = 10 mg in 0.5 mL of solvent. + soluble at RT, +/- soluble at 40 °C, - insoluble. [b] [C] = 50 mg in 10 mL of DMSO at 30 °C.

Thermal properties of polymers

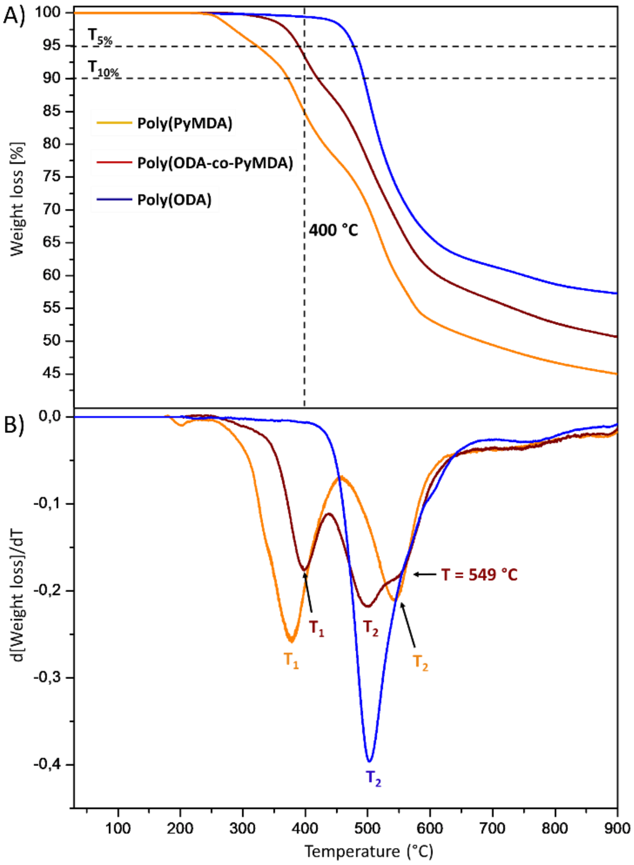

Figure S4. TGA (A) and DTGA (B) thermograms of poly(PyMDA), poly(PyMDA-co-ODA) and poly(ODA).

Table S2. Polymers thermal properties

polymers	T_i(°C)	$T_{5\%}$(°C)	$T_{10\%}$(°C)	T_1(°C)	T_2(°C)	Tg(°C)	R(%)
poly(PyMDA)	328	344	368	374	539	254	44
poly(PyMDA-co-ODA)	358	389	420	397	495	278	49
poly(ODA)	467	479	495	[a]	499	[a]	56

T_i: onset temperature, $T_{5\%}$ y $T_{10\%}$: loss temperature of 5 % and 10 % mass, T_1 y T_2: maximum decomposition rate temperatures, Tg: glass transition temperature, R: waste percentage, [a]: no loss of mass or transitions.

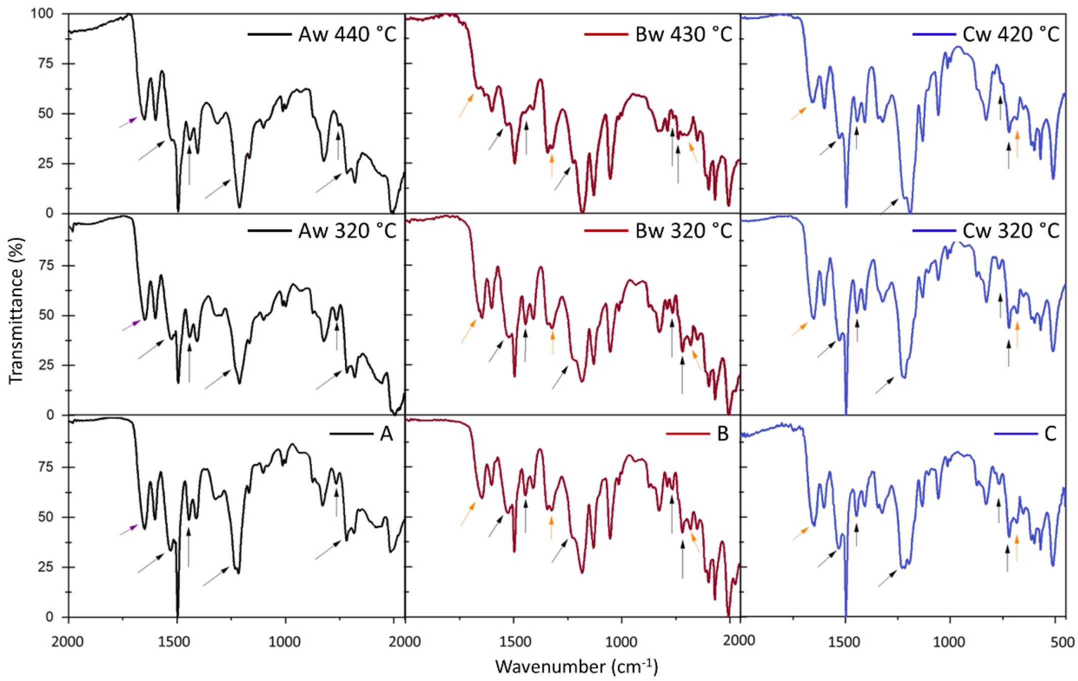

Figure S5. Comparison of FT-IR spectra of poly(ODA-co-PyMDA) (A), poly(ODA-co-PyMDA [Me]) TFSI⁻ (B) and poly (ODA-co-PyMDA [Et]) TFSI⁻ (C) with its waste (poly(ODA-co-PyMDA) waste (Aw), poly(ODA-co-PyMDA [Me])TFSI⁻ waste, (Bw) and poly(ODA-co-PyMDA [Et])TFSI⁻ waste (Cw)) after heating to 420 and 320 °C

Contact angle

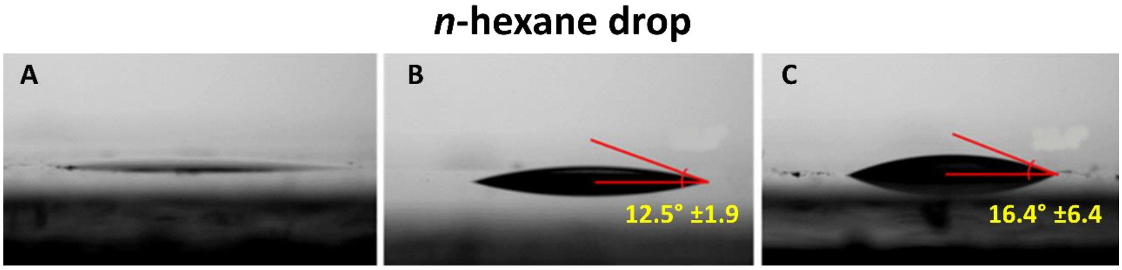

Figure S6. Images of *n*-heptane drop on poly(ODA-co-PyMDA) (A), poly (ODA-co-PyMDA [Me]) TFSI⁻ (B) and poly (ODA-co-PyMDA [Et]) TFSI⁻ (C)
